# Antimicrobial Potential of Natural Compounds of Zingiberaceae Plants and their Synthetic Analogues: A Scoping Review of *In vitro* and *In silico* Approaches

**DOI:** 10.2174/0115680266294573240328050629

**Published:** 2024-04-04

**Authors:** Kok-Hou Yit, Zamirah Zainal-Abidin

**Affiliations:** 1 Department of Craniofacial Diagnostics & Biosciences, Faculty of Dentistry, Universiti Kebangsaan Malaysia, 50300, Kuala Lumpur, Malaysia

**Keywords:** Antimicrobial, Bioactive compounds, Synthetic derivatives, Zingiberaceae, *In vitro*, *In silico*

## Abstract

**Aims:**

There has been increased scientific interest in bioactive compounds and their synthetic derivatives to promote the development of antimicrobial agents that could be used sustainably and overcome antibiotic resistance.

**Methods:**

We conducted this scoping review to collect evidence related to the antimicrobial potential of diverse natural compounds from Zingiberaceae plants and their synthetic derivatives. We followed the Preferred Reporting Items for Systematic Reviews and Meta-Analyses (PRISMA) Extension for Scoping Reviews guidelines. The literature search was conducted using PubMed, Web of Science and Scopus electronic databases for relevant studies published from 2012 to 2023. A total of 28 scientific studies fulfilled the inclusion criteria. The authors of these studies implemented *in vitro* and *in silico* methods to examine the antimicrobial potency and underlying mechanisms of the investigated compounds.

**Results:**

The evidence elucidates the antimicrobial activity of natural secondary metabolites from Zingiberaceae species and their synthetic derivatives against a broad panel of gram-positive and gram-negative bacteria, fungi and viruses.

**Conclusion:**

To date, researchers have proposed the application of bioactive compounds derived from Zingiberaceae plants and their synthetic analogues as antimicrobial agents. Nevertheless, more investigations are required to ascertain their efficacy and to broaden their commercial applicability.

## 1 INTRODUCTION

Herbs have been utilised for medical and culinary purposes for thousands of years. Recent advancements necessitate increased research efforts to identify pharmacologically active herbal plants and their bioactive compounds for phytotherapy [1]. Due to their historical use as medicines, plants of Zingiberaceae (the ginger family) are gaining popularity among the public. Zingiberaceae is the most prominent family within the order Zingiberales; its members include diverse bioactive constituents with great ethnopharmacological value [2]. This family covers more than 50 genera, with 1300 species, and is widespread in tropical and subtropical climates, with the most remarkable diversity in South and Southeast Asia [3, 4]. Several genera of this family, including *Alpinia*, *Zingiber*, *Curcuma*, *Hedychium*, *Amomum*, *Elettaria* and *Kaempferia*, are renowned for their medicinal properties, as exemplified by Javanese ginger (*Curcuma xanthorrhiza* D.Dietr.), turmeric (*Curcuma longa* L.), galangal (*Alpinia galanga* L.) and ginger (*Zingiber officinale* Roscoe) [2].

The aromatic flowering plants in this family exhibit horizontal or creeping tuberous rhizomes and are globally applied as perennial herbs for various purposes. Indeed, due to their aromatic odours and pungent characteristics, these plants have served extensively as spices and/or flavouring agents. The rhizomes are characterised by their capacity to segment and display sympodial branching. The colour of the rhizomes can distinguish distinct species, ranging from pale yellow to dark yellow, greenish-blue, pink or a combination of these colours [5]. The rhizomes usually harbour leaves arranged in a transverse or parallel fashion, with morphologically varied structures, shapes and sizes. The Zingiberaceae plants mainly express small or large labellum, lateral staminodes, narrow and long filaments, and unilocular or trilocular ovaries [5]. In the following paragraphs, we introduce several genera and species of Zingiberaceae, including their purported therapeutic purposes, putative biological activity and bioactive constituents.

**Fig. (1):**
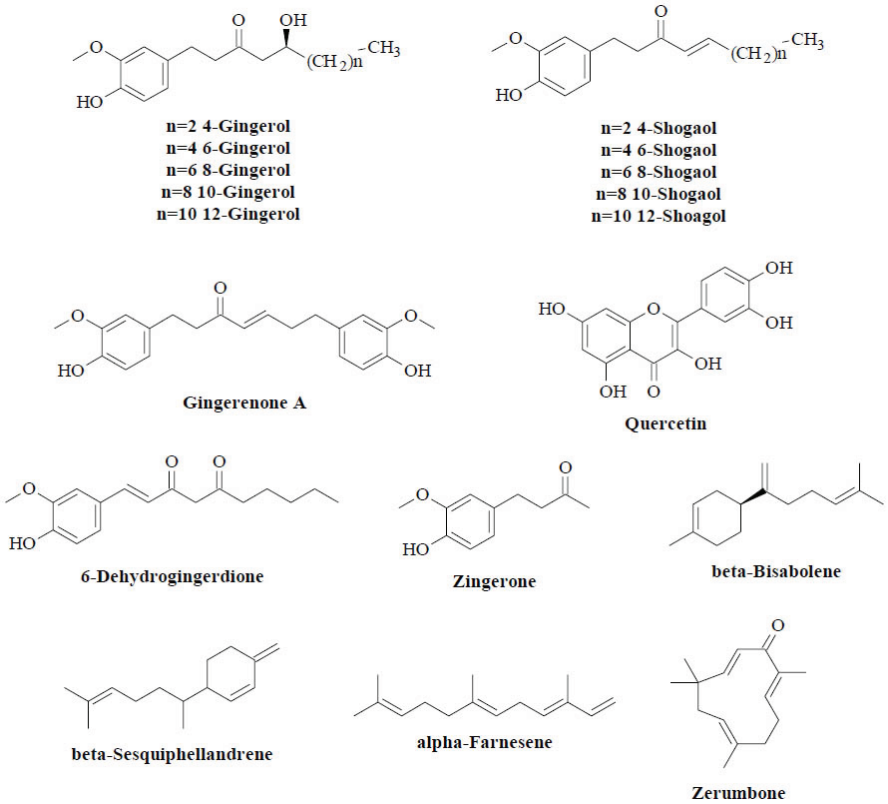
Chemical constituents of the genus *Zingiber*.

The rhizomes of *Zingiber* species are particularly favoured in traditional healing systems [6]. *Zingiber* species contain a plethora of chemical compounds, including organic acids, flavonoids, gingerols, diarylheptanoids, volatile oils and terpenoids (Fig. **1**) [7]. *Z. officinale* is a common ginger from the *Zingiber* genus, rich in phenolic and terpene phytochemicals [6, 8]. Gingerols, including 4-, 6-, 8-, 10- and 12-gingerol, are the major pungent phenolic compounds in fresh ginger rhizomes [9]. These gingerol-related compounds are thermally labile and prone to dehydration. Thus, they can be converted to the corresponding shogaol analogues that give dried ginger its pungent flavour [10]. Other than those reported main compounds, gingerenone A, quercetin, 6-dehydrogingerdione and zingerone are also the phenolic phytochemicals in ginger. In contrast, terpenes such as zingiberene, beta-bisabolene, beta-sesquiphellandrene and alpha-farnesene are the most prevalent components of ginger essential oil [11]. These compounds have antimicrobial properties [12-14]. Another *Zingiber* species, *Zingiber zerumbet* (L.) Sm., has been thoroughly studied for its essential oil, the key compound of which is zerumbone [15]. This compound has several promising pharmacological properties, including antibacterial and antioxidant capabilities [16, 17].

**Fig. (2):**
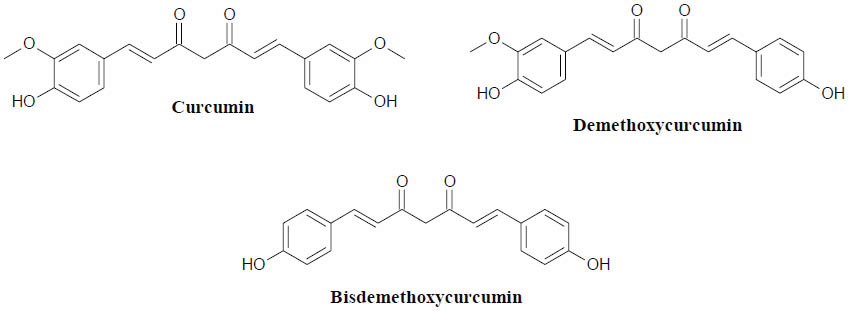
Chemical constituents of the genus *Curcuma*.

*Curcuma* species have been used for centuries to address discrete health complications. The major bioactive components of *Curcuma* rhizomes are non-volatile curcuminoids, as well as volatile sesquiterpenoids and monoterpenoids (Fig. **2**). Curcuminoids include curcumin, demethoxycurcumin and bisdemethoxycurcumin. Both demethoxycurcumin and bisdemethoxycurcumin are curcumin analogues, and these three compounds are the polyphenols responsible for the yellow pigmentation of turmeric rhizomes [18]. On the other hand, sesquiterpenoids and monoterpenoids are the main classes of compounds found in essential oils. *Curcuma* essential oils have been promoted as ideal candidates for manufacturing pharmaceutical and cosmetic products due to their antioxidant and antimicrobial properties [19]. *C. longa*, commonly referred to as turmeric, has a wide range of pharmacological activities - including anticancer and anti-inflammatory - due to its high curcumin content [20, 21].

**Fig. (3):**
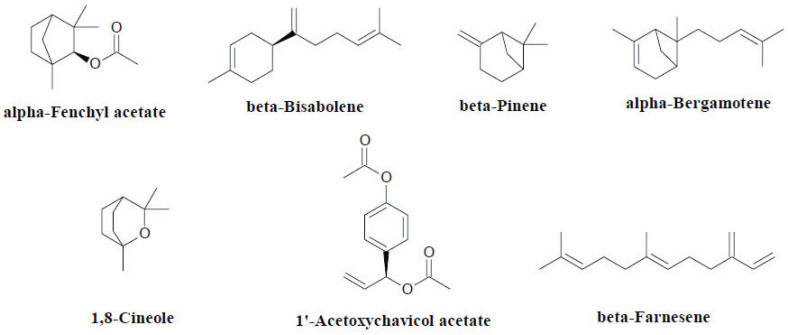
Chemical constituents of the genus *Alpinia*.

*Alpinia*, has been investigated for its biological activities, including combating pathogenic microbes and treating severe infections and diseases. Phytochemical analysis has indicated the presence of diarylheptanoids, terpenes including sesquiterpenoids and monoterpenes, and flavonoids in this genus (Fig. **3**). These compounds possess anti-inflammatory, hepatoprotective, antioxidant and anticancer effects [22]. There are five characteristic subtypes of diarylheptanoids, which are structurally distinct phenolic compounds, in this genus: linear, dimeric, cyclic, chalcone/flavanone and novel [23]. Linear and chalcone/flavanone diarylheptanoids are commonly found in *Alpinia* species. Most of the research on this genus has focused on *Alpinia galanga* (L.) Willd. due to the higher number of bioactive compounds it contains compared with the other species of the genus, including alpha-fenchyl acetate, beta-bisabolene, beta-pinene, alpha-bergamotene, 1,8-cineole, 1′-acetoxychavicol acetate and beta-farnesene [24]. The essential oil derived from dried and fresh *A. galanga* has shown to exert antimicrobial effects on various bacteria, yeast, fungi and parasitic organisms [25].

**Fig. (4):**
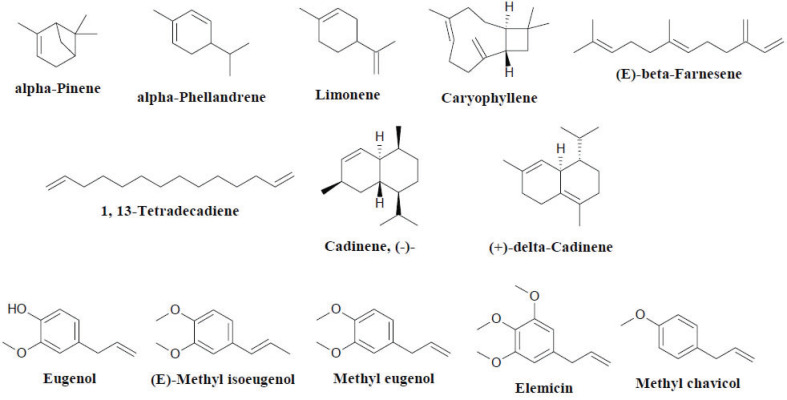
Chemical constituents of the genus *Etlingera*.

The members of *Etlingera* also synthesise essential oils. Alpa-pinene, alpha-phellandrene, beta-pinene and limonene are the monoterpenes present in the essential oils; meanwhile, the major sesquiterpenes are caryophyllene, (E)-beta- farnesene, 1,13-tetradecadiene and cadinene (Fig. **4**) [26].

Phenylpropanoids, often characterised as the prominent organic compounds in *Etlingera* volatile oils, are derived from phenylalanine and tyrosine precursors through the shikimic acid pathway. The presence of phenylpropanoids allows the plants to cope with biotic and abiotic stresses. Eugenol, (E)-methyl isoeugenol, methyl eugenol, elemicin and methyl chavicol are the most researched phenylpropanoids in *Etlingera* species [26]. *Etlingera elatior* (Jack) R.M.Sm. is widely cultivated in Southeast Asia and substantially applied for decorative and culinary purposes in Malaysian and Thai communities [27]. *Etlingera coccinea* (Blume) S.Sakai & Nagam., which is endemic to Borneo, has been used to treat gastrointestinal complications in folk medicine [27]. The essential oils derived from *Etlingera fimbriobracteata* (K.Schum.) R.M.Sm [28], *E. elatior* [29], *Etlingera sayapensis* A.D.Poulsen & Ibrahim [30] and *Etlingera pavieana* (Pierre ex Gagnep.) R.M.Sm [31], as well as the crude extracts from *E. coccinea* [32], *Etlingera sessilanthera* R.M.Sm [32]. and *E. elatior* [33], have been assessed regarding their antibacterial properties against a variety of gram-positive and gram-negative bacteria; they have demonstrated moderate to strong antibacterial activity.

**Fig. (5):**
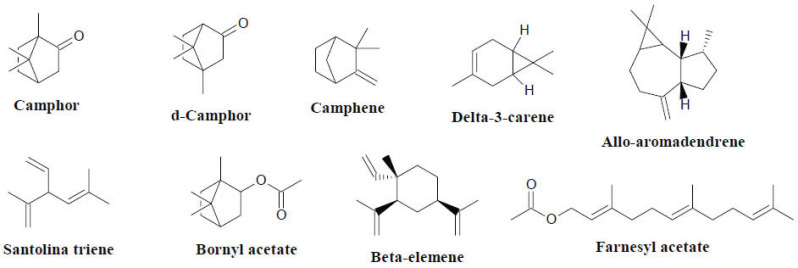
Chemical constituents of the genus *Amomum*.

Plants of the *Amomum* genus have traditionally been applied to treat stomach problems, oral infections, respiratory diseases, malaria, cancer and inflammatory conditions [34, 35]. The *Amomum* genus has been explored for its principal compounds, such as flavonoids, terpenoids and diarylheptanoids, and up to 160 compounds have been isolated and their biological activities have been discussed (Fig. **5**) [34]. These efficacious phytochemicals contribute to their antimicrobial, antioxidant and antiallergic properties [34]. Furthermore, the phytochemical profiles of *Amomum* volatile oils have revealed the limonene, 1,8-cineole, camphor, alpha-pinene, caryophyllene, santolina triene, bornyl acetate, beta-elemene, delta-3-carene, allo-aromadendrene, farnesyl acetate, methyl chavicol, d-camphor, beta-pinene and camphene as the major compounds [35]. *Amomum subulatum* Roxb., usually referred to as black cardamom or the ‘Queen of Spices’, is one of the essential commercial crops of the Himalayan region and is exploited for its medicinal values in managing various respiratory illnesses [36].

**Fig. (6):**
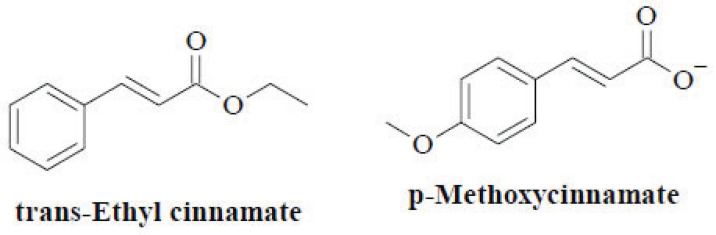
Chemical constituents of the genus *Kaempferia*.

In traditional Asian medicine, the *Kaempferia* genus has been proved to cure distinct ailments, such as wound infection, cough, infectious diseases, and gastrointestinal disorders [37]. The plants of this genus contain the usual secondary metabolites of the other Zingiberaceae genera, such as phenolic compounds, flavonoids, diterpenoids and volatile oils (Fig. **6**). The isopimarane-type diterpenoids are the most abundant in this genus with inspected anti-inflammatory activity, whereas steroids are a minor class of natural compounds in *Kaempferia* species [38]. The essential oils derived from this species comprise phenylpropanoids and cinnamates as the main secondary metabolites, followed by monoterpenes. *Kaempferia galanga* L., the representative species in this genus, possesses pharmacological and curative functions attributed to *trans*-ethyl cinnamate and *p*-methoxycinnamate in the extracted essential oils [39]. Hence, owing to the presence of various constituent classes in Kaempferia volatile oils, they are especially involved in antimicrobial and antioxidant activities [40].

**Fig. (7):**
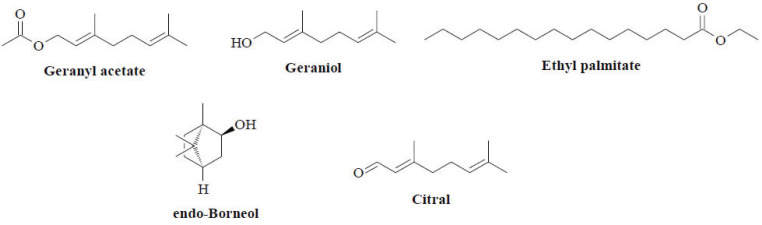
Chemical constituents of the genus *Distichochlamys*.

*Distichochlamys* M.F.Newman is a small ginger genus uniquely endemic to Vietnam. To date, only four species belonging to this genus have been discovered: *Distichochlamys benenica* Q.B.Nguyen & Škornicˇk., *Distichochlamys citrea* M.F.Newman, *Distichochlamys orlowii* K.Larsen & M.F.Newman and *Distichochlamys rubrostriata* W.J.Kress & Rehse [41]. *Distichochlamys citrea* has been conventionally involved in traditional medicinal systems, serving to ameliorate inflammation and infection-associated clinical conditions or as a natural antibacterial agent [42]. Geranyl acetate, geraniol, ethyl palmitate and endo-borneol are extracted from *D. citrea* rhizomes with *n*-hexane (Fig. **7**) [43]. Both geraniol and geranyl acetate are monoterpenes, granting the antimicrobial properties of the *D. citrea* extracts. Regarding essential oils, analogues of 1,8-cineole and citral are the key components reported in *D. citrea*, while geranyl acetate, beta-pinene, beta-elemene and beta-caryophyllene mostly constitute *D. orlowii* volatile oil [44].

The antimicrobial activity of Zingiberaceae plant extracts and essential oils has been critically reviewed. However, there has not been a comprehensive scoping review discussing the antimicrobial properties of the individual plant-derived or synthetic bioactive compounds from Zingiberaceae plants. Increasing evidence-based scientific knowledge regarding compounds with antimicrobial properties can ensure the development and use of medicinal plant-based products. Therefore, in this review, we discuss the characteristics of relevant studies that involve the Zingiberaceae species; the types of studies; the compounds analysed; the methods used for the detection, isolation, characterisation or synthesis of compounds; and the assessment of antibacterial, antifungal and antiviral activities.

## 2 METHODS

### 2.1 Eligibility Criteria

This review only includes published studies. We selected studies in English that discussed the antibacterial, antifungal and/or antiviral activities of natural biologically active compounds from Zingiberaceae plants and their synthetic derivatives against human pathogenic microbes.

### 2.2 Information Sources

We searched the Web of Science, Scopus and PubMed databases for suitable articles published from 2012 to 2023. The primary reason for limiting the search period was to obtain up-to-date evidence on the antimicrobial properties of natural bioactive compounds and related synthetic analogues.

### 2.3 Search Strategy

In the first stage, we used suitable keywords - ‘Bioactive compound’, ‘Zingiberaceae’ and ‘Antimicrobial’ with suitable Boolean operators AND/OR and truncation symbol $ - to search the selected databases (Table **1**).

### 2.4 Study Selection Process

We excluded several studies from the search results, including review articles, case reports, conference proceedings, protocol papers and book reviews. Duplicate records were eliminated with the help of EndNote version 20. Moreover, we screened the titles and abstracts of the publications and assessed them by using the inclusion criteria to select the content that could answer the objectives. Referring to the selected titles and abstracts, the full-text articles were critically appraised to determine which articles could be used for ongoing analysis and included in this scoping review.

### 2.5 Data Charting

In the third stage, we extracted the necessary information from the chosen articles. Two authors independently examined the derived data, and any discrepancies were resolved through a consensus discussion. A data-charting form was created in Excel. The two authors reviewed the findings and revised the data charting form.

### 2.6 Data Items

The data extracted from each article included the authors, year of publication, study type, Zingiberaceae species, compounds, methodology, bacterial/fungal/viral species, antibacterial/antifungal/antiviral activity, and study outcomes as sorted out in the supplementary material (Tables **S1-S3**). The study types were *in vitro*, *in silico* or a combination of both. We authenticated the taxonomic information of the Zingiberaceae species by using the World Flora Online (WFO) database. The methodology involved compound isolation, synthesis and characterisation, and antimicrobial assays. The two authors only included compounds with positive antimicrobial activity for *in vitro* studies or those that displayed notable binding energy and documented interaction with targets for *in silico* research.

## 3 RESULTS

### 3.1 Selection of Sources of Evidence

As shown in Fig. (**8**), our database searches yielded 317 titles. There were two duplicates, so we screened the title and abstract of 315 records for eligibility, whereby 277 studies were omitted, leaving 38 articles. Two studies could not be retrieved; thus, 36 articles progressed to the subsequent comprehensive full-text review. Finally, we extracted data from 28 articles that satisfied the inclusion criteria and answered the research question of this scoping review. The rationale for the exclusion of the eight articles was: three solely discussed crude extracts, essential oils or bioactive fractions but not individual compounds; one used undefined reporting units of antibacterial and antifungal activities; one did not mention the antimicrobial assay performed; one used phytopathogen as the study target; one reported the synergistic effect of the compound with other agents, without testing a single compound; and one reported negative results of the tested compounds.

### 3.2 General Characteristics of the Included Studies

Out of the included 28 articles, 18 studies involved only *in vitro* experiments, 7 discussed only *in silico* analyses and 3 conducted both *in vitro* and *silico* analyses. Eight studies were carried out in India, four in Malaysia, four in Indonesia, two in China, two in the United Kingdom, two in Japan, two in the Republic of Korea, and one each in Saudi Arabia, Iran, Brazil and Vietnam.

All plants evaluated in the included studies are part of the Zingiberaceae family. Fig. (**9**) shows the distribution of the Zingiberaceae plant species involved in the included studies. *Z. officinale* (12 studies) was the most studied species, followed by *Z. zerumbet* (3 studies), *C. longa* (3 studies), *Curcuma caesia* Roxb. (2 studies), *Alpinia conchigera* Griff. (1 study), *Alpinia purpurata* (Vieill.) K.Schum (1 study), *D. benenica* (1 study), *Zingiber montanum* (J. Koenig) A.Dietr. (1 study), *Etlingera pubescens* (B.L.Burtt & R.M.Sm.) R.M.Sm. (1 study), *Kaempferia pandurata* Roxb. (1 study), *Alpinia mutica* Roxb. (1 study), *Alpinia zerumbet* (Pers.) B.L.Burtt & R.M.Sm. (1 study), *Amomum tsao-ko* Crevost & Lemariaé (1 study) and *Amomum nilgiricum* V.P.Thomas & M.Sabu (1 study).

The efficacy of Zingiberaceae plant compounds as antimicrobial agents has been assessed against a broad panel of microorganisms: gram-positive and gram-negative bacteria with or without drug-resistance characteristics, fungi and viruses. As depicted in Fig. (**10**), the included studies tested 31 distinct species of microorganisms (17 bacteria, 11 fungi and 3 viruses), comprising reference strains, clinical strains and *in silico* models. *Staphylococcus aureus* (a gram-positive bacterium), *Escherichia coli* (a gram-negative bacterium), *Candida* spp. (fungi), *Aspergillus* spp. (fungi) and severe acute respiratory syndrome coronavirus 2 (SARS-CoV-2) are the major target microorganisms of the studied compounds.

**Fig. (11):**
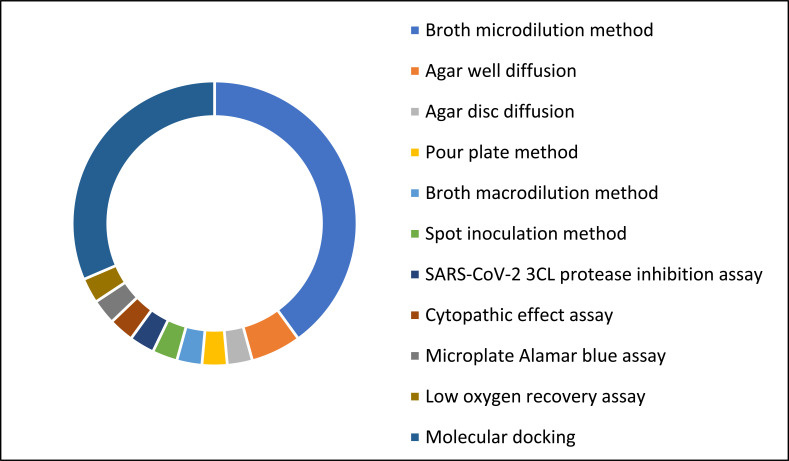
Methods used for the assessment of antimicrobial activities.

The included studies employed various methods to evaluate the antimicrobial potential of natural or synthetically produced compounds from Zingiberaceae plants (Fig. **11**).

The broth microdilution method was the most prominent assay and the gold-standard reference method (14 studies) for determining the minimum inhibitory concentration (MIC) and the minimum bactericidal concentration (MBC) as indicators of antibacterial and antifungal properties. Several studies utilised diffusion tests, including the agar well (2 studies) and agar disk diffusion (1 study) techniques, to determine the inhibition zone as part of the screening test. The authors conducted *in silico* molecular docking simulations to investigate the interaction between phytochemicals and binding sites in bacteria and fungi and to propose the possible underlying antibacterial and antifungal mechanisms of those compounds (4 studies). Other methods, such as broth macrodilution (1 study), spot inoculation (1 study), the pour plate method (1 study), the microplate Alamar blue assay (1 study) and the low oxygen recovery assay (1 study) were used to determine antibacterial and antifungal activities.

To appraise the antiviral properties of plant compounds, the studies most often used the molecular docking approach for *in silico* analyses (7 studies), with SARS-CoV-2 serving as the examined target. For the *in vitro* study of bioactive compounds against SARS-CoV-2, a 3CL protease inhibition assay (1 study) was performed. The cytopathic effect (CPE) assay (1 study) was also used to elucidate viral inhibition by compounds *in vitro*.

### 3.3 Assessment of the Main Study Outcomes

To investigate the antibacterial effects exerted by selective natural or synthetic compounds, 17 studies reported the activity against diverse species and strains. Seven of these studies explained the contribution of the chemical structures of the compounds to their antibacterial activity [45-51]. Two studies reported that the antibacterial activity of purified compounds was superior to those of plant extracts and essential oils, even though the authors hypothesised that there is a synergistic interaction between the plethora of compounds identified through phytochemical screening [52, 53]. Two studies revealed that the compounds were less effective against gram-negative bacteria than against gram-positive bacteria [46, 54], and one study disclosed the inability of the compound to inhibit the involved gram-negative bacteria with the tested concentration range [55]. One study demonstrated the synergistic antibacterial effects of plant compounds and antibiotics [56].

Six studies evaluated the antifungal properties of active compounds. One study related antifungal activity to the structure of the compounds [45]. One study reported that the killing effects of the compound on fungal strains were more effective than the commercial antifungal agent [57]. One study demonstrated the enhanced antifungal effect of a plant-derived compound on another natural antifungal agent [58]. In addition, one study reported that a single isolated compound had a superior antifungal effect than rhizome essential oils [53]. The antiviral potential of plant compounds has been evaluated against several structural targets of SARS-CoV-2, including 3C-like protease (3CL^pro^) or main protease (M^pro^) [59-61], papain-like protease PL^pro^ [61] and spike protein [62-67], showing the binding interaction between the compounds and target sites of SARS-CoV-2 *via* an *in silico* approach.

### 3.4 Antibacterial Properties

The authors of the included studies have unravelled the antibacterial activity of bioactive compounds and related synthetic analogues and have elucidated some mechanisms of action. Pham *et al.* [54] discovered *trans*-cinnamic acid (**1**), borneol (**2**) and *trans*-*o*-coumaric acid (**3**) with successive column chromatography from *n*-hexane extract of *D. benenica*. *Trans*-*o*-coumaric acid (**3**) and *trans*-cinnamic acid (**1**) inhibited the growth of gram-positive *S. aureus* and *Bacillus subtilis* (MIC = 1.52-3.37 mM) better than borneol (**2**) (MIC = 6.49 mM). In contrast, the three compounds showed low activity against the gram-negative bacteria *Pseudomonas aeruginosa* and *E. coli* (MIC = 6.1-6.75 mM).

**Scheme 1:**
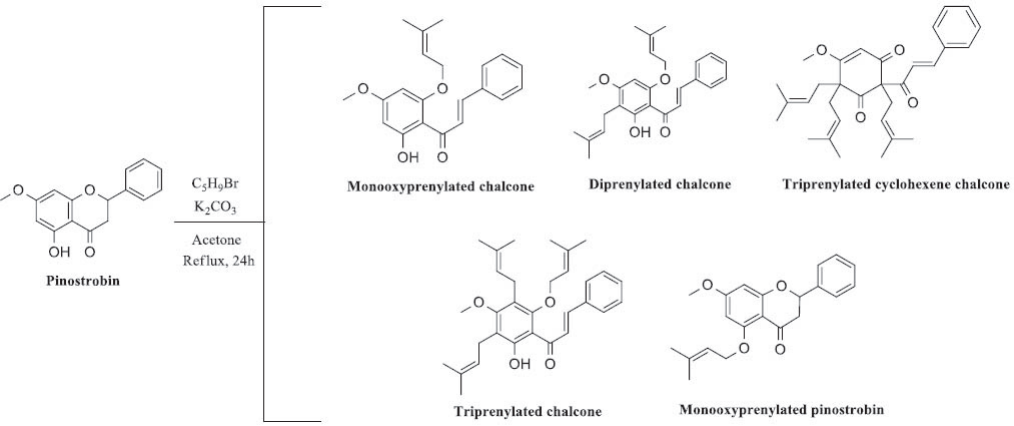
Synthesis of pinostrobin derivatives.

Marliyana *et al.* [47] evaluated the antibacterial potential of pinostrobin (**4**) from *K. pandurata* and its five derivatives transformed through prenylation, namely monooxyprenylated chalcone (**5**), diprenylated chalcone (**6**), triprenylated cyclohexene chalcone (**7**), triprenylated chalcone (**8**) and monooxyprenylated pinostrobin (**9**). After synthesis using pinostrobin (**4**) as the starting material (Scheme **1**), the analogues exhibited significantly better antibacterial activity (MIC = 25-50 µg/mL) compared with their precursor (MIC = 37.5-150 µg/mL). The authors claimed that the flavanone ring in pinostrobin (**4**) substituted with prenyl groups could increase their lipophilic properties and thus improve their ability to pass through the bacterial membrane and their antibacterial activity.

Mohamad Taib *et al.* [68] obtained 1′S-1′-acetoxychavicol acetate (**10**) from *A. conchigera* rhizome with excellent antimicrobial activity (MIC = 0.5 mg/mL) against methicillin-resistant *S. aureus* (MRSA). However, *trans*-*p*-coumaryl diacetate (**11**), the major compound of *A. conchigera*, could be chemically transformed from 1′S-1′-acetoxychavicol acetate (**10**) through [3, 3]-sigmatropic rearrangement and only showed moderate activity against the same strain (MIC = 1.0 mg/mL). Auranamide (**12**), a novel phenylalanine derivative isolated from the leaf of *A. mutica*, had an inhibitory effect on *E. coli*, *P. aeruginosa*, *S. aureus* and *B. subtilis* (MIC = 500 µg/mL) when compared with the derived essential oil and *n*-hexane extract (MIC = 1000 µg/mL) [69].

**Scheme 2:**
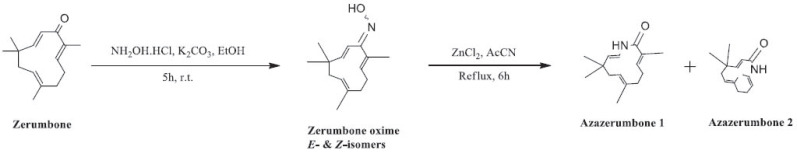
Synthesis of zerumbone derivatives.

Working with MRSA and multidrug-resistant *S. aureus*, Siddique *et al.* [50] isolated the terpenes (*E*)-8(17),12-labdadiene-15,16- dial (**13**) and zerumbol (**14**) from *Z. montanum*; they showed significant antibacterial activity (MIC = 32-128 µg/mL, 0.145-0.291 mM). (*E*)-8(17),12-labdadiene-15,16-dial (**13**) contains an exomethylene at C-8, two aldehyde groups at C-16 and C-17, as well as an olefine at C-12, which the authors attributed to the compound’s antibacterial activity against antibiotic-resistant strains. Correspondingly, zerumbol (**14**) synthesised from zerumbone (**15**) was the most effective antibacterial agent (MIC = 50-125 ppm) against *Bacillus cereus*, *S. aureus*, *E. coli* and *Yersinia enterocolitica* compared with its precursor and other synthetic derivatives such as zerumbone oxime (**16**), azazerumbone 1 (**17**) and azazerumbone 2 (**18**) (Scheme **2**) [49]. The authors speculated that the chemically modified carbonyl moiety enhances the antibacterial activity of zerumbol (**14**). Azazerumbone 2 (**18**), with an amide group in the 12-membered carbon ring, also demonstrated better antibacterial activity than the precursor zerumbone (**15**), unveiling the structure-activity relationship for those compounds.

Tian *et al.* [53] reported that purified zerumbone (**15**) obtained through recrystallisation had the most effective antibacterial effects against *Enterococcus faecalis*, *S. aureus*, *B. subtilis*, *E. coli*, *P. aeruginosa* and *Proteus vulgaris*, with inhibition zones of 9.48-15.72 mm at 100 mg/mL and a MIC of 32.25-250.00 µg/mL compared with fresh and dry *Z. zerumbet* essential oils. Likewise, zerumbone (**15**) possessed anti-*Helicobacter pylori* activity with a remarkable MIC of 250 µg/mL. It also showed gastroprotective efficacy in an *in vivo* model of *H. pylori*-induced peptic ulcers [70].

**Scheme 3:**
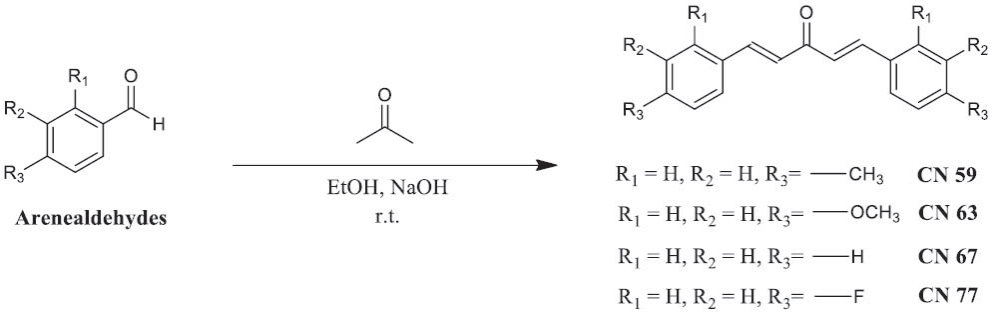
Synthesis of monocurcuminoids.

Cerveira *et al.* [71] reported the antibacterial properties of curcumin (**25**) extracted from *C. longa* powder and its synthetic monocarbonyl analogues CN 59 (**26**), CN 63 (**27**), CN 67 (**28**) and CN 77 (**29**) (Scheme **3**) against six microbial strains (MIC = 4.06-150 µg/mL). Interestingly, a combination of CN 77 (**29**) and turmeric powder exerted a synergistic antibacterial effect on *Aeromonas hydrophila* and *P. aeruginosa* with a fractional inhibitory concentration (FIC) index of not more than 0.5.

**Scheme 4:**
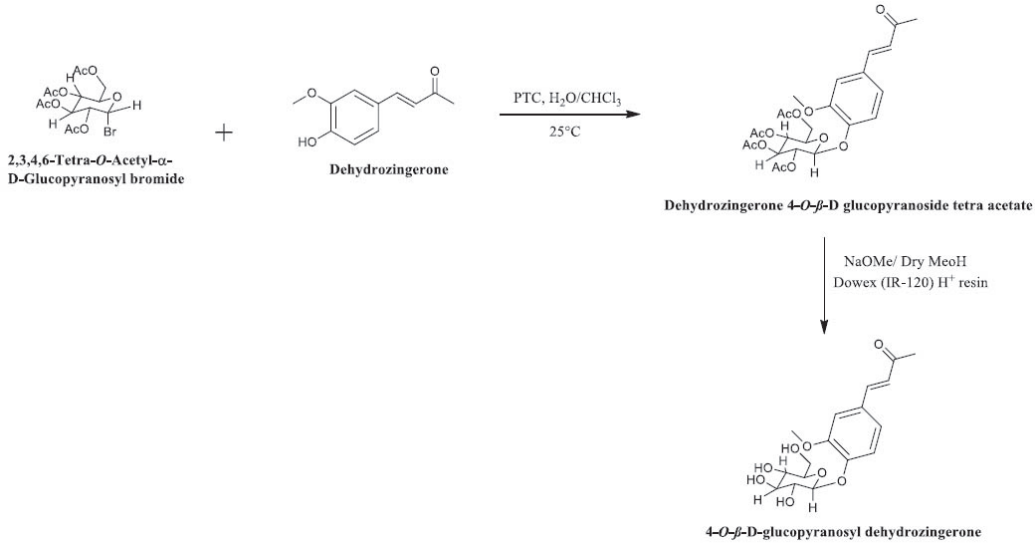
Synthesis of dehydrozingerone derivatives.

Kubra *et al.* [45] studied food-borne pathogenic bacterial species, including *Listeria monocytogenes*, *B. subtilis*, *Salmonella enterica* serotype Typhi and *E. coli*. They synthesised dehydrozingerone (**19**), a phenolic compound that can be naturally isolated from *Z. officinale* rhizomes, and its four derivatives - dehydrozingerone 4-*O*-*ß*-d-glucopyranoside tetra acetate (**20**), 4-*O*-*ß*-d-glucopyranosyl dehydrozingerone (**21**), 4-*O*-acetyl dehydrozingerone (**22**) and methyl ether of dehydrozingerone (**23**) (Scheme **4**) - and tested their effects against the aforementioned species by implementing the agar well diffusion method. All the included compounds displayed moderate to strong inhibitory effects against the bacterial species thanks to the *α*,*β*-unsaturated carbonyl (C = O) group in their chemical structures. The researchers explained that dehydrozingerone (**19**) works similarly to the antibacterial mechanism of action of vanillin by targeting the cytoplasmic membrane, negatively influencing ion gradients and pH homeostasis.

In another study, researchers isolated a diarylheptanoid named etlingerin (**24**) from *E. pubescens* and reported its bactericidal effect against *S. aureus*, *B. cereus* and *B. subtilis* (MIC = 0.0625-0.125 mg/mL; MBC = 0.0625-0.125 mg/mL) [55]. The SYTO-9/propidium iodide uptake method indicated the detrimental effect of this compound on the bacterial membrane: it promoted the leakage of cellular content such as DNA and proteins based on the bacterial cellular leakage assay.

*C. caesia* Roxb. yielded the phenolic compound curcumin (**25**), the major curcuminoid present in turmeric, based on bioassay-guided isolation from crude methanolic root extract. The broth macrodilution sensitivity test showed that compared with the crude extract, curcumin exhibited lower MICs and MBCs against a panel of gram-positive *Staphylococcus* and *Bacillus* strains (MIC = 0.0625-0.25 mg/mL) and gram-negative *K. pneumoniae*, *E. coli*, *P. aeruginosa* and *Proteus vulgaris* (MIC = 0.25-1 mg/mL) [52].

Focussing on *Z. officinale*, two representative phenolic compounds - 6-gingerol (**30**) and its dehydrated product 6-shogaol (**31**) - showed moderate to potent antibacterial activity against gram-positive *E. faecalis*, *B. subtilis* and effluxing MRSA strains (MIC = 8-512 mg/L) and the gram-negative bacteria *P. aeruginosa*, *E. coli*, *K. pneumoniae* and *Proteus* sp. (MIC = 128-512 mg/L) [48]. The lower activity against gram-negative bacterial species could be due to the permeability barrier to the antibacterial compounds or unfavourable structural modification of the compounds to reduce their activity. Nevertheless, the aliphatic side chain in 6-gingerol (**30**) and the presence of a double bond in 6-shogaol (**31**) account for their effective antibacterial activity. In the same study employing the broth mating method, the researchers noted that the anti-plasmid activity of 6-gingerol (**30**) and 6-shogaol (**31**) actively hindered the transfer of resistant plasmids in *E. coli* (TP114, PUB 307 and PKM 101) that is facilitated by bacterial type IV secretion system, suggesting their potential as natural anti-plasmid agents that assist in overcoming antibiotic resistance.

**Scheme 5:**
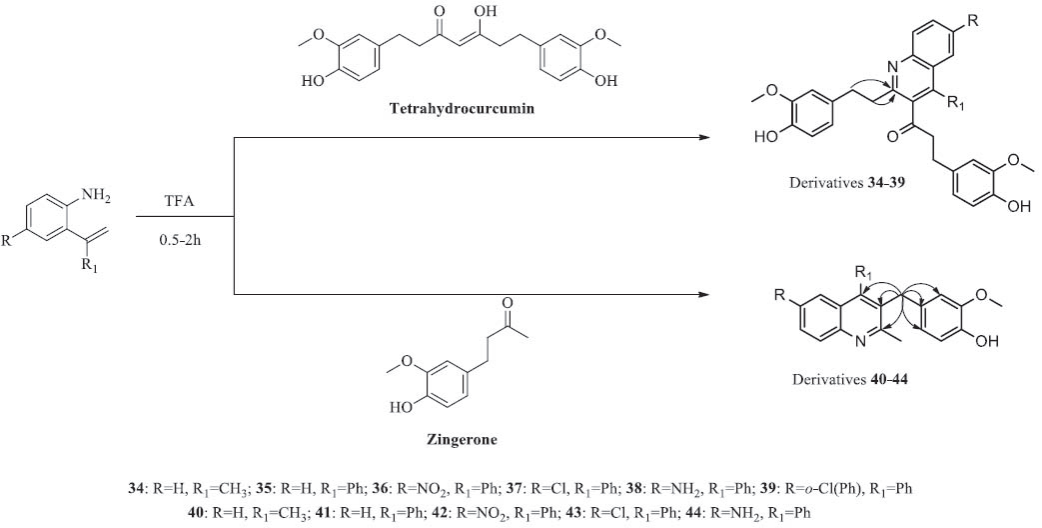
Synthesis of quinoline derivatives from tetrahydrocurcumin and zingerone.

Two structurally similar natural compounds, zingerone (**32**) and tetrahydrocurcumin (**33**) from *Z. officinale* and *C. longa* harbour a carbonyl moiety and 4-hydroxy-3-me- thoxyphenyl group, respectively. In addition to these naturally occurring bioactive compounds, Manjunatha *et al.* [46] evaluated the antibacterial activity of quinoline derivatives (**34-44**) synthesised with the aid of the catalyst trifluoroacetic acid (Scheme **5**) against *S. aureus*, *B. cereus*, *Y. enterocolitica* and *E. coli*. Their findings suggested that the quinoline derivatives (**34-44**), with either 2-aminoacetophenone or 2-aminobenzophenone substitution at the carbonyl group, ameliorated antibacterial properties with lower MICs than tetrahydrocurcumin (**33**) and zingerone (**32**).

Mehta *et al.* [56] reported the strong antibacterial activity of lariciresinol (**45**) from *Z. officinale* against AcrAB-TolC *S. enterica* serovar Typhimurium strains *in vitro* (MIC = 400 µg/mL). The group later found a synergistic effect of this compound with tetracycline, lowering the MIC of this antibiotic by 2-4-fold against these pathogenic strains with an efflux pump. Further, *in silico* molecular docking indicated that lariciresinol (**45**) could target the TetR 61E9 (RamR) transcriptional repressor and the 61E8 (RamA) transcriptional activator with stronger affinity (-8.2 and -7.4 kcal/mol, respectively) that chenodeoxycholic acid and tetracycline. Lariciresinol (**45**) also adhered to Lipinski’s rule of five - it fulfilled the drug-likeness criteria and was non-toxic in the toxicity prediction analysis - and thus could be recommended as a natural efflux pump inhibitor for *S. enterica* ser. Typhimurium to manage drug-resistant infection.

Lee *et al.* [51] successfully isolated nine aliphatic compounds, including tsaokol B (**46**), (*E*)-2-decene-1,10-diol (**47**), tsaokol A (**48**), acetoxytsaokol A (**49**), (2*E*,8*E*)-2,8-decadiene-1,10-diol (**50**), (2*E*,6*E*)-1,8-diacetoxy-2,6-octadiene (**51**), (*E*)-decenal (**52**), (*E*)-dec 2-enyl acetate (**53**), (*E*)-2-dodecen-1-yl acetate (**54**), and 3 acyclic terpenoids of geraniol (**55**), geranyl acetate (**56**) and (3*R*)-(*E*)-nerolidol (**57**) from *A. tsao-ko*. These compounds presented an antimycobacterial effect against replicating (MIC = 0.6 to >100 µg/mL) and non-replicating (MIC = 1.4 to >100 µg/mL) *Mycobacterium tuberculosis* H37Rv. Among the compounds, tsaokol A (**48**) demonstrated the greatest inhibitory effects on the tested strains with the presence of an olefinic hydrocarbon at the C-8 position.

Computer-aided molecular docking revealed that among the 25 identified compounds in *A. nilgiricum*, serverogenin acetate (**58**) stood out as the best binding ligand (-103.14 kcal/mol) with bacterial target protein 5iwm, which is a DNA gyrase subunit [72]. This compound forms a hydrogen bond with Gly341. However, an *in vitro* assessment should be conducted to confirm this compound’s antibacterial efficacy and underlying mechanism of action.

Khuntia *et al.* [73] examined the antibacterial effects of six active constituents from *C. caesia* essential oils against drug-resistant *E. coli* and *S. aureus* strains. The enlisted compounds were muurola-4-10(14)-dien-1-ol (**59**), globulol (**60**), viridiflorol (**61**), germacrone (**62**), epicurzerenone (**63**), curzerenone (**64**), eucalyptol (**65**) and camphor (**66**). Curzerenone (**64**) showed the best antibacterial activity against *E. coli* and *S. aureus* by producing the largest inhibition zones (10 and 11 mm, respectively) and the lowest MICs (3.125 and 1.56 µg/mL, respectively). *In silico* molecular docking indicated the lowest binding energy for viridiflorol (**61**) with -9.02 kcal/mol against tyrosyl-tRNA synthetase as the antibacterial target of *S. aureus* by hydrogen bonding with AspA40 and TyrA170 residues, thus suggesting its ability to interfere with bacterial protein synthesis. On the other hand, muurola-4-10(14)-dien-1-ol (**59**) interacted with glucosamine 6-phosphate synthase of *E. coli* at SerA174 with a binding energy of -6.09 kcal/mol. This enzyme is important in cell wall biosynthesis.

### 3.5 Antifungal Properties

*In vitro* and *in silico* studies have been carried out to assess the antifungal properties of natural compounds from Zingiberaceae species and their synthetic derivatives to illuminate the structure-function relationship and to resolve the complex mechanisms of action. The authors synthesised dehydrozingerone (**19**) from *Z. officinale* and its derivatives (**20-23**) *via* stepwise processes and examined their antifungal activity against *Fusarim* sp. Gt-1019, *Penicillium* sp., *Aspergillus flavus*, *Aspergillus ochraceus*, *Aspergillus oryzae* and *Aspergillus niger* by using the agar diffusion method; the inhibition zone was 15.0-31.5 mm at a dose of 1 mg [45]. The authors stated that the carbonyl group confers antifungal efficacy to the compounds. The polarisation of the carbon-oxygen double bond in the aldehyde or keto group likely explains their ability to form covalent bonds with fungal DNA and proteins and to disrupt fungal metabolism.

Based on *in silico* molecular docking, serverogenin acetate (**58**) derived from *A. nilgiricum* also has antifungal potential [72]. Among the 25 detected compounds in gas chromatography-mass spectrometry analysis, serverogenin acetate (**58**) showed the lowest binding energy with the fungal target 4i9p (-105.78 kcal/mol) and displayed the best phytoligand-protein complex binding stability *via* hydrogen bonds with Thr170, Arg173, Ser504, Lys503, His502 and His469.

Kim and Eom [57] interrogated the species-dependent antifungal effect of 6-shogaol (**31**) from *Z. officinale*. They applied this pungent ginger compound to several *Candida* clinical isolates, specifically *Candida auris*, *Candida glabrata* and *Candida tropicalis*. Apart from the intense inhibitory activity against the planktonic form of these fungi at 50% and 80% of the population (MIC_50_ = 16 to >64 µg/mL; MIC_80_ = 32 to >64 µg/mL), the compound also showed inhibitory and eradication effects of 80% of *C. auris* biofilm matrix at low concentrations (MBIC80 = 32-64 µg/mL; MBEC80 = 32 µg/mL). Confocal laser scanning microscopy of the pre-formed *C. auris* biofilms treated with 6-shogaol (**31**) revealed reduced levels of proteins, nucleic acids and carbohydrates in the biomass as well as a low cell density. The authors demonstrated the fungicidal properties of this compound in a time-kill assay, with a dose-dependent decrease in the viability of the fungal strains. Most importantly, the compound could decrease the expression of the *CDR1* gene in *C. auris*, which is associated with efflux pump activity. Hence, this compound might be useful in clinical settings to address resistant fungal infection [57].

Tian *et al.* [53] evaluated the antifungal activity of zerumbone (**15**) derived from *Z. zerumbet* against *Candida albicans*. The MIC and MBC were 31.25 and 250.00 µg/mL, respectively. Besides, 100 mg/mL zerumbone produced an inhibition zone of 11.31 ± 0.83 mm on the agar inoculated with *C. albicans*. This sesquiterpenoid is an active antifungal compound and mainly contributes to the antifungal activity of *Z. zerumbet* essential oil.

Cerveira *et al.* [71] sought to determine the inhibitory and eradication effects of the naturally occurring phenolic compound curcumin (**25**) and its monocurcuminoid derivatives (**26-29**) against *C. albicans*. The antifungal activity of the structural variants (**26-29**) was based on the targeted modification of the methylene bridge in curcumin (**25**). The derivatives showed a fungistatic effect: CN 59 (**26**) (MIC = 4.06 µg/mL; MBC = 16.25 µg/mL), CN 63 (**27**) (MIC = 4.69 µg/mL; MBC = 18.75 µg/mL), CN 67 (**28**) (MIC = 60 µg/mL; MBC = 240 µg/mL) and CN 77 (**29**) (MIC = 140 µg/mL; MBC = not within the concentration range). The authors also elucidated the synergistic fungal inhibitory effect of monocurcuminoid CN 77 (**29**) and turmeric powder on *C. albicans* (FIC index ≤ 0.5) in the checkerboard assay. They proposed using low doses of each agent to ameliorate *C. albicans*-mediated fungal infections.

Khuntia *et al.* [73] investigated the inhibitory effects of globulol (**60**) and epicurzerenone (**63**) on *C. albicans*. Among the eight tested compounds (**59-66**), the lowest MIC was 12.5 µg/mL. Furthermore, the molecular docking results supported the *in vitro* antifungal effect of globulol (**60**): a stable complex with *N*-myristoyl transferase *via* a hydrogen bond at LeuA355 had the lowest binding energy (-8.43 kcal/mol). Targeting this protein could inhibit fatty acid translocation and disrupt the membrane integrity of *C. albicans*.

Aisy *et al.* [74] presented the antifungal potential of stigmasta-5,22-dien-3-ol, acetate, (3.beta.) (**67**) and farnesyl acetate (**68**) from *A. purpurata* by means of molecular docking. Stigmasta-5,22-dien-3-ol, acetate, (3.beta.) formed a stable complex with lanosterol-14-α-demethylase of *Candida krusei* by binding to His377 and Phe380 in the active site with a binding energy of -13.53 kcal/mol, while farnesyl acetate interacted with these residues with a binding energy of -7.15 kcal/mol. Both docked compounds complied with Lipinski’s rule of five, indicating their bioavailability and potential use as antifungal agents that disrupt the fungal cell membrane.

Yamano *et al.* [58] showed that dehydrozingerone (**19**) from *Z. officinale* improved the antifungal effects of glabridin, an isoflavane derived from the *Glycyrrhiza glabra* L. roots, against *C. albicans* and *Saccharomyces cerevisiae* in the checkerboard assay. The combined treatment markedly reduced the expression of the *PDR1*, *PDR3* and *PDR5* genes - which encode efflux pumps in *S. cerevisiae* - compared with glabridin alone. This compound also indirectly altered the translation of Pdr5p, an ABC transporter. Overall, these findings provide evidence for the use of combination therapy involving plant-derived compounds against drug-resistant fungi.

### 3.6 Antiviral Properties

In response to the coronavirus disease 2019 (COVID-19) pandemic, since 2020, researchers have employed *in silico* molecular docking in computer-aided drug design to evaluate many phytoligands identified from Zingiberaceae species as potential antiviral agents against SARS-CoV-2. Zubair *et al.* [60] proposed combining chromatographic/spectroscopic methods with docking analysis and molecular dynamics simulations to identify the potential antiviral bioactive compounds originating from *Z. officinale*. The authors found that three steroid compounds - 24-methylcholesta-7-en-3*β*-on (**69**) (-68.80 kcal/mol), spinasterol (**70**) (-78.11 kcal/mol) and spinasterone (**71**) (-87.4 kcal/mol) - have a lower binding energy and a stronger binding affinity to the SARS-CoV-2 3CL protease. The 3CL protease represents a promising therapeutic target due to its role in regulating coronavirus replication. In the molecular docking analysis, the authors discussed several notable interactions between the compounds and 3CL protease amino acids, including a hydrogen bond between the hydroxyl group of spinasterol (**70**) and Thr190; a hydrogen bond between the carboxyl group of 24-methylcholesta-7-en-3β-on (**69**) and Cys44; and hydrophobic interaction between spinasterone (**71**) and Val42, Leu167, Cys44 and Pro168. The authors subsequently investigated the *in vitro* inhibitory effect of 24-methylcholesta-7-en-3*β*-on (**69**) extracted from ginger pseudostem on SARS-CoV-2 3CL^pro^ by performing an inhibition assay; there was 75% enzymatic inhibition at a concentration of 500 µM. The subsequent molecular dynamics simulation showed that 24-methylcholesta-7-en-3*β*-on (**69**) could bind to His41 and Cys145 in the catalytic site with a corresponding root mean square fluctuation (RMSF) of 0.755 and 0.880 Å, respectively. These findings underscore the inhibitory mechanism of 24-methylcholesta-7-en-3*β*-on (**69**) at the molecular level.

Wijaya *et al.* [66] determined that the phenolic compound 4-gingerol (**72**) from *Z. officinale* can target the SARS-CoV-2 main protease (also termed M^pro^ or 3CL^pro^). This compound showed the lowest binding energy (-7.3 kcal/mol) among the 16 investigated compounds and could interact with the main protease domains by hydrophobic bonding at Gln110, Phe294, Asn151 and Val104, as well as hydrogen bonding at Thr111, Asn151 and Asp153. As mentioned by the authors, the formation of hydrogen bonds is vital to facilitate ligand-protein interactions, protein folding and breakdown. Moreover, this compound did not violate Lipinski’s rule of five, and the molecular dynamics simulation showed a stable ligand-protein complex conformation with an RMSF below 1-3 Å.

In a comprehensive study, Mehmood *et al.* [59] chose six distinct medicinal plants originating from different families to identify compounds with antiviral properties against SARS-CoV-2. Based on the docking analysis, the researchers highlighted a monoterpene (**73**) from *Z. officinale* because it interacted with the inhibitory drug targets RNA-dependent RNA polymerase (RdRP) that governs coronavirus replication, 3CL^pro^ and angiotensin-converting enzyme 2 (ACE2) as the binding site of SARS-CoV-2 spike protein for subsequent replication. The authors selected this monoterpene (**73**) for docking analysis because it fulfilled Lipinski’s rule of five, had previously reported antiviral activity, complied with absorption, distribution, metabolism, excretion and toxicity (ADMET) pharmacokinetic properties, and had been subjected to drug toxicity prediction. It formed hydrogen bonds with Ser15 and alkyl bonds with Met87 and Lys411, with a binding energy of -4.7 kcal/mol. This monoterpene (**73**) could form a hydrogen bond with Gly110 and an alkyl bond with Lys152 of SARS-CoV-2 3CL^pro^, with a binding energy of -6.4 kcal/mol. Nevertheless, when targeting the ACE2 protein, only alkyl bonding was observed with Ile106 and Lys103, and the binding energy was -5.6 kcal/mol.

Umashankar *et al.* [67] deciphered the potential natural bioactive anti-SARS-CoV-2 compounds from eight traditional Indian medicinal plants and a vast number of phytochemical moieties. The authors included compounds from *Z. officinale* and *C. longa* in the docking and simulation analysis. Among the compounds from different libraries, geraniin (**74**) (-8.2 kcal/mol) from *Z. officinale* and *O*-demethylde- methoxycurcumin (**75**) (-8 kcal/mol) from *C. longa* displayed a suitable binding energy through the formation of hydrogen bonds and hydrophobic interactions with the favourable residues on receptor binding domain (RBD) of spike glycoprotein, particularly the glycosylation sites. Viral glycosylation plays a role in viral evasion and replication, thus contributing to the pathogenesis of SARS-CoV-2 in the human body [75, 76]. Because the complex formed between the viral spike protein and *O*-demethyldemethoxycurcumin (**75**) was more stable (ligand root mean square deviation (RMSD) of 1.0-2.5 Å) and exhibited prominent ligand interactions, thus the authors considered it the best-hit compound. The intermolecular interactions between the compound and the hotspots of the ACE2 binding site and the residues of the glycosylation site on the RBD as well as the optimal conformation during the simulation process, indicated its function as a dual-acting inhibitory drug against ACE2 interactions and glycosylation of spike protein.

**Fig. (12):**
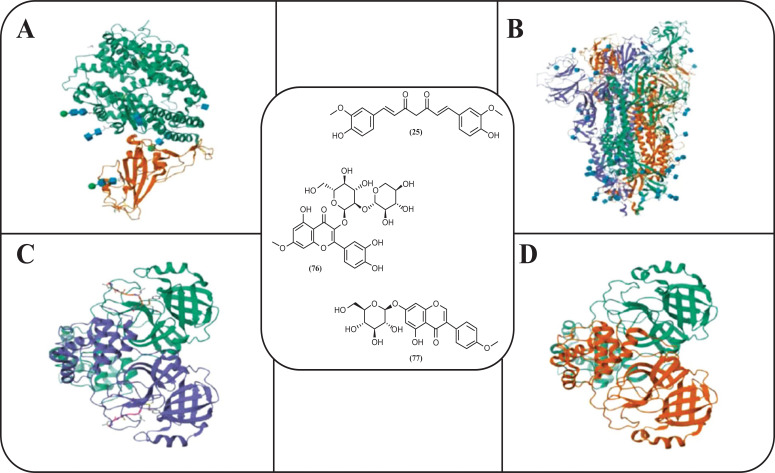
Three compounds from *Zingiber officinale* Roscoe were docked to the SARS-CoV-2 targets with prominent interactions, which included (**A**) the RBD-ACE2 complex (PDB: 6VW1), (**B**) pre-fusion 2019-nCoV spike glycoprotein with a single receptor-binding domain (PDB: 6VSB) and two M^pro^ structures, namely (**C**) PDB: 6LU7 and (**D**) PDB: 6M03 [62].

Babaeekhou *et al.* [62] identified dereplicated bioactive compounds from *Z. officinale via* molecular networking and employed molecular docking to estimate their binding affinity to three SARS-CoV-2 targets (Fig. **12**). The included targets were RBD-ACE2 complex (PDB: 6VW1) [63], spike glycoprotein with the RBD (PDB: 6VSB) [64] and M^pro^ protein structures (PDB: 6LU7 and PDB: 6M03) [65]. From the virtual analysis, a flavonoid named 3-[(2S,3R,4S,5S,6R)-4, 5-dihydroxy-6-(hydroxymethyl)-3-[(2S,3R,4S,5R)-3,4,5-trihydroxyoxan-2-yl] oxyoxan-2-yl]oxy-2-(3,4-dihydroxyph- enyl)-5-hydroxy-7-methoxychromen-4-one (**76**) (-9.27 kcal/mol) and sissostrin (**77**) (-8.643 kcal/mol) had the highest affinity scores for 6VW1, while curcumin (**25**) (-10.126 kcal/mol) presented the best docking score for 6M03. The flavonoid (**76**) also exhibited significant binding affinity to 6VSB (-9.96 kcal/mol), and the docking analysis for 6LU7 indicated the lowest binding energy (-9.399 kcal/mol) for sissostrin (**77**). The authors postulated that hydrogen bonds, pi-pi interactions, and ionic bonds between phytoligands and the viral binding sites could be the mechanisms for inhibiting viral entry and replication. Hence, novel natural drugs might represent a way to manage SARS-CoV-2 infections.

Al-Sanea *et al.* [61] were interested in combining *Z. officinale* methanolic extract with silver nanoparticles (AgNPs) as an antiviral drug. The authors used the *in vitro* 3-(4,5-dimethylthiazol-2-yl)-2,5-diphenyltetrazolium bromide (MTT) assay to assess the anti-SARS-CoV-2 activity. The biosynthesised AgNPs with ginger methanolic extract (median inhibitory concentration [IC_50_] = 0.034 µg/mL) had greater antiviral activity than the ginger methanolic extract alone (IC_50_ = 206.4 mg/mL). Metabolomic profiling of ginger methanolic extract revealed 15 compounds. Three of these compounds - quercetin (**78**), 5,6-epoxycholestan-3-ol (**79**) and riboflavin (**80**) - achieved relatively comparable docking scores to the assigned co-crystallised ligand of nonstructural protein 16 (NSP16) and adaptor-associated kinase 1 (AAK1). NSP 16, also known as 2′-*O*-Mtase, helps SARS-CoV-2 to evade the immune response by catalysing 2′-*O*-methylation of SARS-CoV-2 RNA and interfering with the induction of type-1 interferon [77, 78]. AAK1, a human enzyme, regulates the SARS-CoV-2 life cycle, including endocytosis, assembly and release of virions [79, 80]. In the comparative molecular docking analysis, quercetin (**78**) is only bound to NSP 16 *via* Ala6914, Gly6946 and Leu6898. Furthermore, the NSP 16 residues Gly6869/6871, Asp6912/6897, Tyr6930, Leu6898, Met6929 and Cys6913 interacted with the three compounds.

**Fig. (13):**
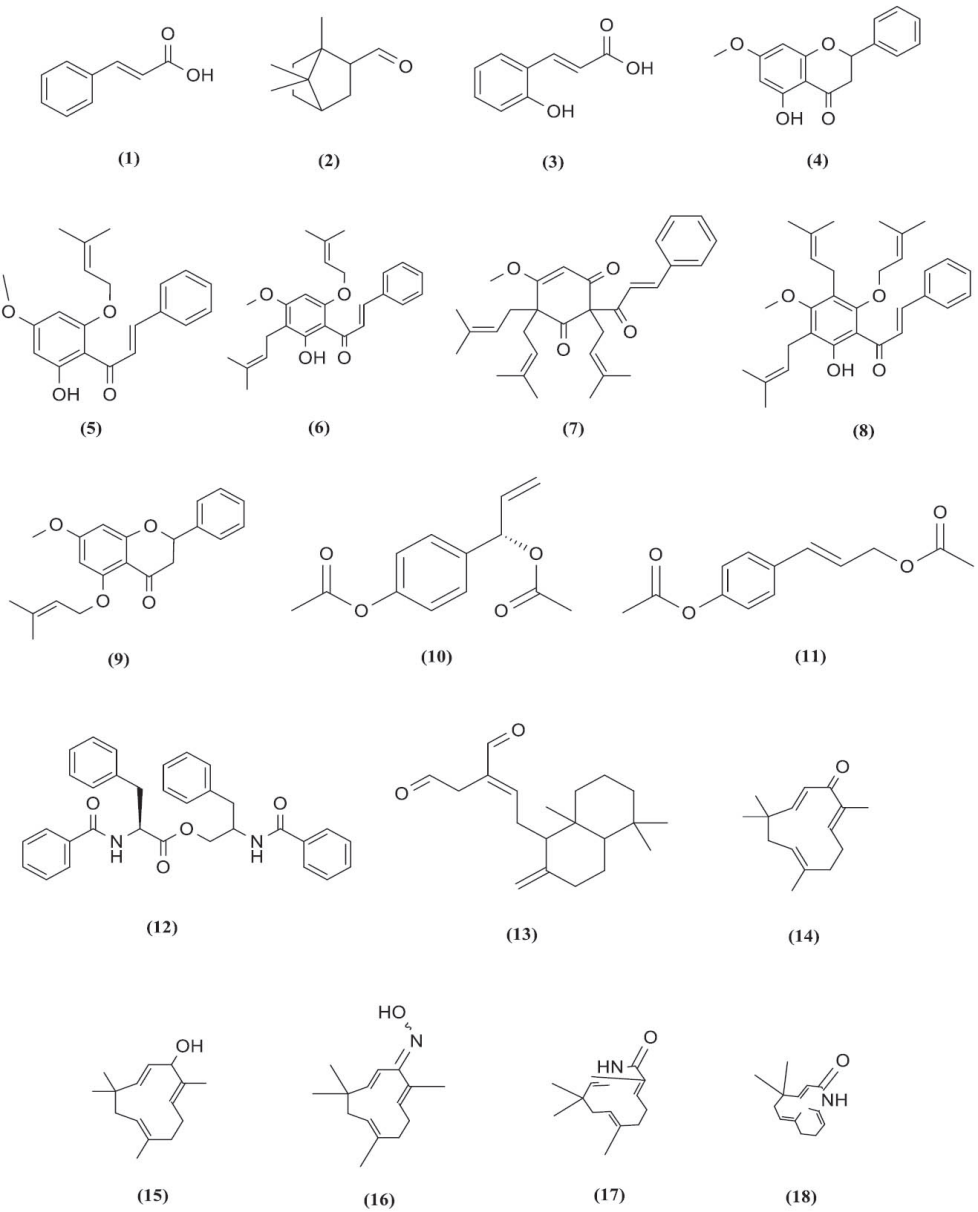
Chemical structures of compounds **1**-**18**.

**Fig. (14):**
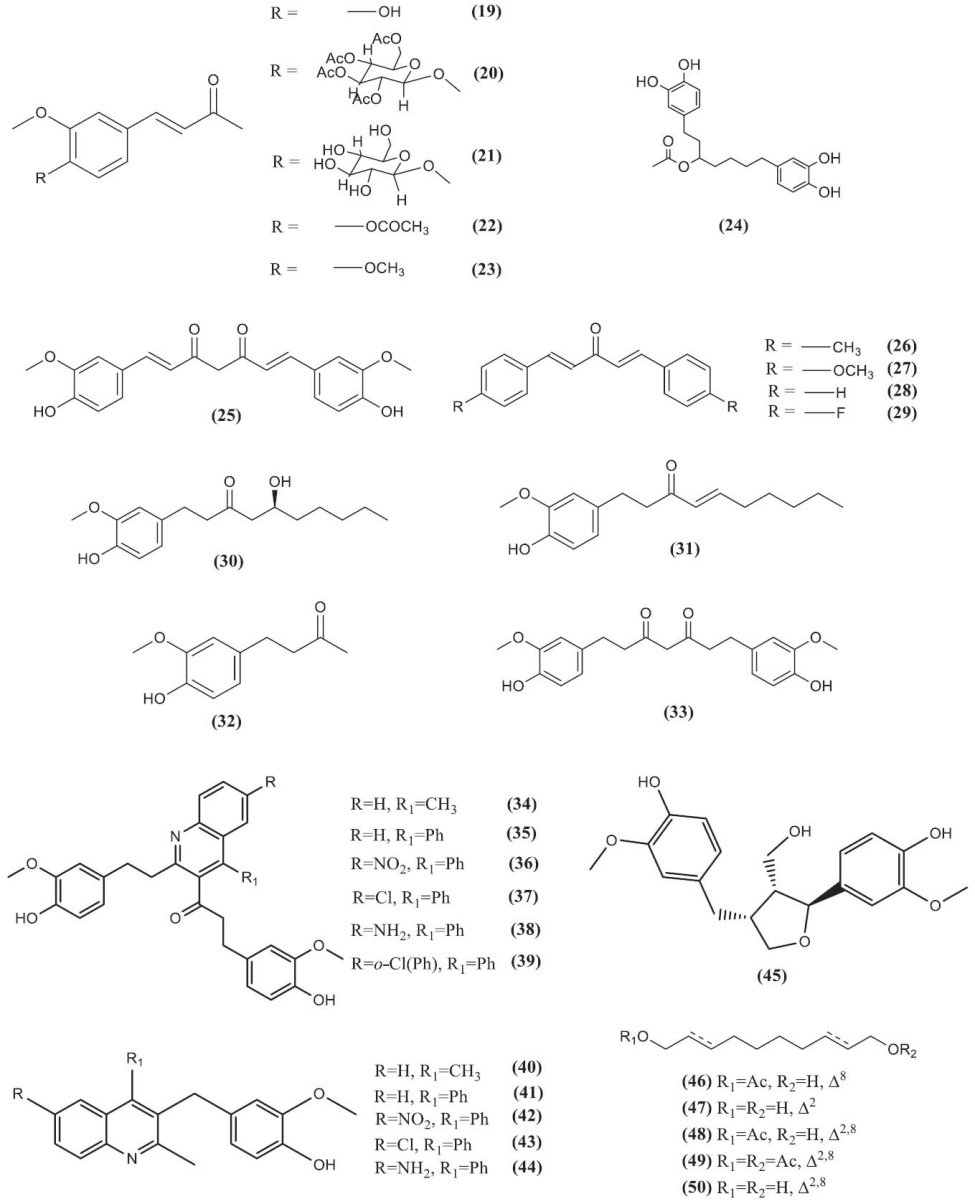
Chemical structures of compounds **19**-**50**.

**Fig. (15):**
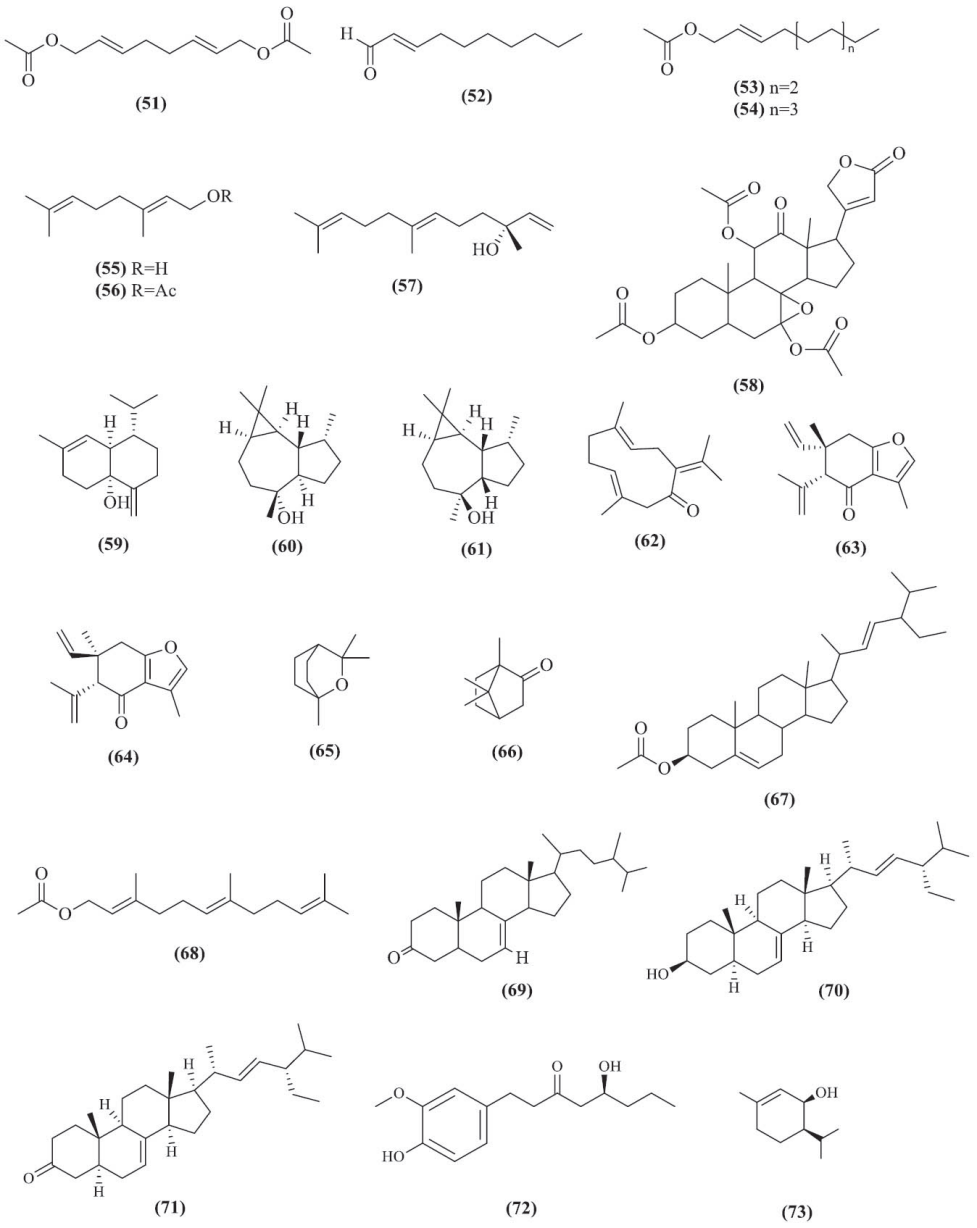
Chemical structures of compounds **51**-**73**.

**Fig. (16):**
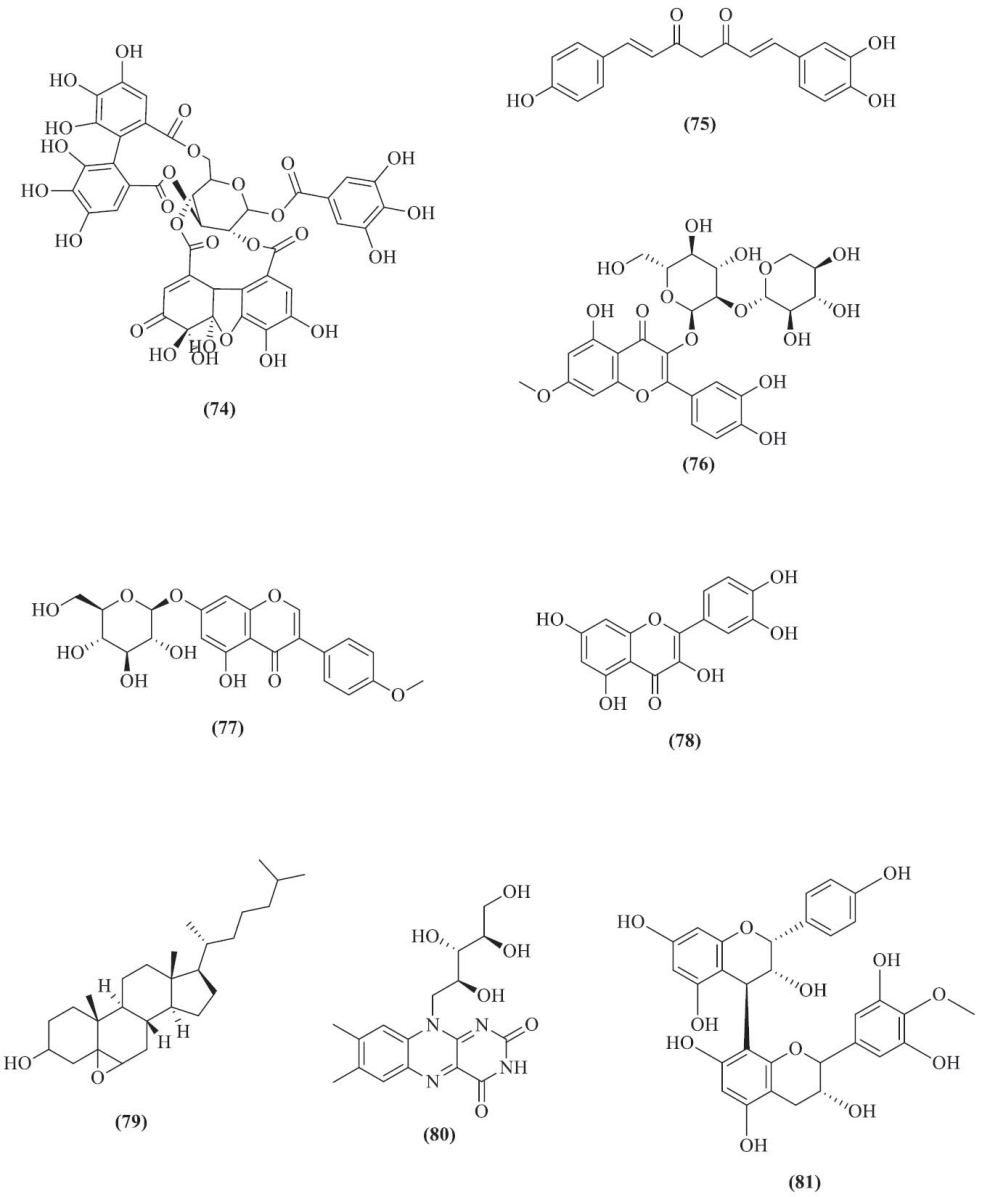
Chemical structures of compounds **74**-**81**.

Other than the SARS-CoV-2 virus, Narusaka *et al.* worked with proanthocyanidins (PACs) (**81**), the major polyphenolic polymers with flavan-3-ols extracted from *Alpinia zerumbet* (Pers.) B.L.Burtt & R.M.Sm., by examining its antiviral effects on the influenza A virus and porcine epidemic diarrhoea virus (PEDV) through the cytopathic effect (CPE) assay [81]. Within the *in vitro* CPE study, the potent antiviral activity of PACs (**81**) against influenza A virus has been uncovered, denoted by decreasing the viral titre by >3 logs at 0.1 mg/mL *via* calculation of 50% endpoint dilution (TCID_50_/mL). Moreover, the PACs (**81**) at 0.1 mg/mL also successfully decreased the viral titre of PEDV, a surrogate for human coronavirus, especially SARS-CoV-2, by >4 logs as compared to the control. PACs (**81**) could effectively inactivate both tested viruses in a dose-dependent manner. In another research work conducted by Konappa *et al.*, the group adopted a molecular docking technique to screen the binding affinity of serverogenin acetate (**58**) on viral target protein 1REV, which is the HIV-1 reverse transcriptase [72]. Interaction between the compound and viral protein through hydrogen-bond formation by Gln91 with the best binding pose and binding energy (-90.53 kcal/mol) has recommended the isolation and purification *via* bioassay-guided fractionation of this compound from *Amomum nilgiricum* V.P.Thomas & M.Sabu for its antiviral potential (Figs. **13**-**16**).

## 4 DISCUSSION

Currently, commercially available antibacterial agents are overused throughout the world to inhibit the growth of pathogenic bacteria. This has resulted in the evolution of resistance mechanisms to various prescription antibiotics, presumably through chromosomal mutations or the transmission of exogenous resistance genes [82, 83]. Over time, an increasing number of multidrug-resistant bacteria inhibit or suppress the antibacterial activity of antibiotics and devastatingly affect their efficacy [84, 85]. Six nosocomial bacterial species are specified in the ESKAPE acronym: the gram-positive bacteria *Enterococcus faecium* and *S. aureus*, and the gram-negative bacteria *K. pneumoniae*, *Acinetobacter baumannii*, *Enterobacter* spp. and *P. aeruginosa*. These species have been commonly described as being highly virulent and exhibiting antibiotic resistance patterns. Considering the studies included in this scoping review, *S. aureus* has been the most researched gram-positive bacteria. Several compounds derived from Zingiberaceae species, including *A. conchigera*, *Z. montanum* and *Z. officinale*, exerted an antibacterial effect on the opportunistic pathogen MRSA [48, 50, 68].

Similarly, a growing number of antifungal drug-resistant strains have resulted in fatal fungal infections, attributable to the widespread use of antifungals for treatment and preventive purposes. This condition is explained by the activation of protective stress response pathways following exposure to antifungals, as opposed to the plasmid-mediated transmission of resistance genes typically observed in the bacterial community [86]. *Aspergillus*, *Cryptococcus*, *Candida* and *Pneumocystis* are the major genera of human pathogenic fungi. Targeted therapy implemented for mucosal or invasive fungal infections caused by these fungi undoubtedly contributes to the development of resistant strains [87]. The data compiled in this review has shown that most of the compounds derived from *Z. officinale* have antifungal effects on *Aspergillus* and *Candida* species [45, 57].

Viruses may deliberately acquire resistance to existing antiviral treatments by undergoing genomic mutations during the replication process [88]. Viruses typically replicate in animals; therefore, exposing animal reservoirs to an environment containing potential antiviral drugs may hasten the establishment of antiviral drug resistance. Some viruses associated with human diseases have been reported to show resistance towards antiviral medications, including human immunodeficiency virus [88], hepatitis B virus [89], influenza virus [90], hepatitis C virus [91] and herpes simplex virus [92, 93]. Intriguingly, oligomers or polymers of monomeric flavan-3-ols named proanthocyanidins (**81**) extracted from *A. zerumbet* could reduce the titre of influenza A virus, indicating the antiviral properties of polyphenolic compounds from plants [81].

Based on the studies included in this review, the scientific community emphasises medicinal herbs or natural plant-derived products with functional phytochemicals as an innovative therapeutic approach to address microbial infections, especially those with resistance issues. Various natural bioactive compounds isolated from plant extracts have been extrapolated and applied in several fields involving the environmental, medical and food industries. Antimicrobial bioactive compounds from herbal medicines can exert growth inhibition or eradication effects on bacteria, fungi, viruses and protozoa *via* distinct mechanisms, as opposed to the currently used antimicrobials; to a certain extent, they may possess relevant therapeutic value in treating diseases caused by resistant microbial strains [94]. The diverse or chemically complex bioactive constituents of plants are relatively more therapeutically effective than manufactured drugs, have fewer adverse effects, and limit the likelihood of developing resistance [95].

The scientific justification for the use of herbal materials and the discovery of novel lead compounds for conventional single chemical entities (SCEs) are the two main foci of ethnopharmacological research in the pharmaceutical sector [96]. Obtaining active fractions from plant extracts through bioassay-guided approaches and the subsequent isolation of pure bioactive compounds are fundamental scholarly and commercial research practices. Therefore, biologically active constituents and/or their derivatives as well as synthetic products based on the chemical structure of these phytochemicals, could all be components of SCEs. Nevertheless, to be approved as an alternative antimicrobial agent, substantial clinical trials must be carried out with a sufficient number of patients with drug-resistant diseases or novel microbial infections. Consideration must be given to thorough *in vivo* research on bioactive compounds with positive antimicrobial activity and minimal toxicity before the validation phase. The use of *in vivo* models to evaluate the antimicrobial activity of pure phytochemicals and structurally modified compounds has yet to be widely adopted [94].

COVID-19 pandemic still represents a human health threat. SARS-CoV-2 is the etiological agent of COVID-19 and has rapidly disseminated around the globe. Currently, no effective and specific therapeutic agent is available for this infectious disease, even though innumerable laboratory and clinical trials have been implemented to seek effective antiviral agents against it [97]. This may be because of the selective effects on specific groups of patients or, in the worst-case scenario, there are little or no therapeutic benefits in patients with COVID-19 in terms of overall mortality, the hospitalisation duration and the requirement for ventilation [98]. Nonetheless, research on the repurposing of existing plant-based compounds, either naturally or synthetically acquired, has been carried out in response to COVID-19 and other emerging viral infections rather than executing *de novo* synthesis of novel antiviral agents. Furthermore, these phytoextracts or derived phytocompounds have been highlighted as emergency medicinal agents during epidemics or pandemics to control infections caused by novel viruses [99].

Based on our literature search, *in vitro* experiments have been implemented most often to investigate the antimicrobial efficacy of compounds from the Zingiberaceae family against bacterial and fungal species, whereas *in silico* methods have been used to study the antiviral potential of the compounds through binding affinity to the target sites, particularly SARS-CoV-2. No information is available regarding the antimicrobial activity of active compounds using *in vivo* models and clinical trials. *in vitro* studies are cell-based experiments that are usually employed in the preliminary stages of screening; however, the biocompatibility of compounds cannot be determined through this type of study. According to the retrieved data, there are substantial variations in the experimental settings for the isolation, stepwise synthesis and antimicrobial testing of compounds from Zingiberaceae plants, thus presenting diverse investigational alternatives for *in vivo* experiments using animal models. Despite their complexity and expense, *in vivo* studies are crucial to elucidate the antimicrobial efficacy and toxicity of potential compounds. Based on our findings, *in vivo* studies and clinical trials that test these Zingiberaceae phytochemicals against microbial infections are needed.

*In silico* studies are usually performed through computer simulations to examine the interaction between natural therapeutic ligands and target receptors, thereby reducing the cost of conducting mass laboratory work and simultaneously accelerating the drug discovery process. This approach relies on high-throughput molecular docking to select the potential hits after narrowing down the search for prospective lead entities from a vast number of compound databases [100]. Lipinski’s rule of five refers to the physicochemical properties of an ideal drug and predicts its drug likeness based on the criteria they should meet: hydrogen bond donors ≤5, hydrogen bond acceptors ≤10, molecular mass <500 Da and partition coefficient (log P) ≤5. As evidenced by the considerable number of studies in this review pertaining to SARS-CoV-2 *in silico* investigations, this technique has been widely attempted to search for potential COVID-19 treatments to alleviate this pandemic. Although the scientific community has been prompted to create *in vitro* and *in vivo* experimental conditions for COVID-19 research, an initial step using *in silico* methods to examine the therapeutic efficacy of diverse plant-based compounds against possible targets of SARS-CoV-2 structures is crucial. The essential targets of SARS-CoV-2 mentioned in the included studies are 3C-like protease (main protease) and papain-like protease, which play a pivotal role in initiating coronavirus replication by forming a functional replicase complex, whereas the spike protein primarily functions in receptor recognition and eases the process of cell membrane fusion [101, 102]. Besides, as SARS-CoV-2 is an RNA virus, a virus-specific enzyme known as RNA-dependent RNA polymerase - which regulates viral genome replication and transcription - could also be a potential therapeutic target [59]. Consequently, the phytoligands with the greatest potential antiviral efficacy, as indicated by negative binding energy to the aforementioned viral targets, could be subjected to *in vitro* and *in vivo* research for further validation.

Based on our search results, compounds from *Z. officinale* have garnered the most interest for their antiviral potential against SARS-CoV-2, either directly towards the viral structures or indirectly by inhibiting viral cellular targets. Another *in silico* study collectively revealed the antibacterial, antifungal and antiviral properties of serverogenin acetate (**58**) from *A. nilgiricum* *via* molecular docking [72]. Serverogenin acetate (**58**) is the only bioactive component included in this review that has been evaluated for antibacterial, antifungal and antiviral activities. It demonstrated favourable ligand-protein interactions with the bacterial 5iwm, fungal 4i9p and viral 1rev target proteins.

Hydrophobic compounds have difficulty crossing the hydrophilic cell wall of gram-negative bacteria, which creates a formidable permeability barrier. On the other hand, hydrophilic compounds are unable to penetrate the inner membrane of gram-negative bacteria, which contains a glycerophospholipid bilayer [103]. As a result, the compounds should have a lower antibacterial efficacy against gram-negative than gram-positive bacteria. However, 6-shogaol (**31**), a hydrophobic ginger polyphenol from *Z. officinale*, had superior antibacterial activity against gram-negative bacteria compared with 6-gingerol (**32**), regardless of the structural barrier of the tested bacteria [48]. This could be due to the presence of a hydroxyl group in 6-gingerol (**32**), which reduces the compound’s lipophilicity and cell membrane permeability, resulting in decreased cell bioavailability [104]. In addition, 6-shogaol (**31**) could inhibit *C. auris* planktonic cells and biofilm in a concentration-dependent manner [57]. Etlingerin (**24**) isolated from *E. pubescens* showed antibacterial activity against *S. aureus* and *B. subtilis* by altering bacterial membrane permeability and, consequently, mediating intracellular leakage in a dose-dependent manner and causing cell death, suggesting the antibacterial mechanism of this compound.

The ability of phytochemicals or their synthetic analogues to exert antimicrobial effects is influenced by their shape, size, structure and chemical properties to reach the targeted site of action. Compounds synthesised from naturally occurring bioactive constituents have modified chemical structures that improve or impair their antimicrobial efficacy. The synthetic variants of zingerone (**40-44**) demonstrated the most potent antibacterial effect against the tested gram-negative bacteria, attributable to the modification of carbonyl functionality in the side chain of its precursor [46]. Other than that, five synthetic derivatives of the bioflavonoid pinostrobin (**5-9**) exhibited enhanced antibacterial activity against a panel of bacterial species, including *S. aureus*, *B. subtilis*, *P. aeruginosa* and *E. coli*, owing to the substitution of the flavone ring system of pinostrobin (**4**) with prenyl groups. This substitution increased the lipophilicity of the analogues, thus facilitating the interaction between compounds and bacterial membranes [47]. Generally, it is necessary to determine how to strategically modify the structure of the compounds, which in turn could improve their affinity and selectivity for the targets, allowing biological activities to take place at the molecular level.

In one study, the authors claimed that zerumbone (**15**) isolated from *Z. zerumbet* showed more effective antibacterial and antifungal activity against the tested bacteria and fungi compared with fresh and dry rhizome essential oils, whereas another study stated that purified curcumin obtained from *C. caesia* exhibited greater antibacterial effect against gram-positive and gram-negative bacteria than the crude extract [52, 53]. Greater antifungal and antibacterial activity of the plant crude extracts, essential oils or fractions may be due to the combined mechanisms of action of numerous classes of bioactive constituents. Nevertheless, their antimicrobial activities were lower than those of the individual isolated compounds in certain studies, suggesting it is necessary to isolate compounds to minimise the underlying antagonistic interactions among the bioactive compounds [105]. Apart from utilising a single product, bioactive constituents from herbal plants or their related synthetic derivatives could be applied in combination with each other or even with commercially available antimicrobial agents to restore their antimicrobial effects and to circumvent the resistance problem [84]. Lariciresinol (**45**), a lignin belonging to phenylpropanoid isolated from *Z. officinale*, produced a synergistic antibacterial effect against *S. enterica* ser. Typhimurium strains when co-administered with a tetracycline antibiotic and lowered the MIC of that antibiotic [56].

There are a few limitations to this scoping review. We included ‘*Zingiber*’ and ‘*Zingiber officinale* Roscoe’ in the search query because this genus includes true gingers within Zingiberaceae and *Z. officinale* is the most well-known garden ginger and a representative of this family [106]. As a result, we included more studies that evaluated *Z. officinale* in this review. Because Zingiberaceae covers a vast array of species and compounds, we included the classes of representative bioactive compounds available in its genera in the keyword search rather than incorporating each individual species, bioactive compound and synthetic analogue into the search. Even though we made every attempt to conduct a thorough search, we may have overlooked certain studies that focused on the bioactive constituents of Zingiberaceae plants or their synthetic derivatives. Moreover, a systematic review and meta-analysis should be conducted to collect the highest level of evidence regarding the antimicrobial potential of compounds isolated from Zingiberaceae members [107]. Despite these limitations, based on the current evidence, natural compounds from the discussed Zingiberaceae species and their synthetic analogues could be alternative agents for the treatment of microbial infections.

## CONCLUSION

Accumulating evidence on the antimicrobial activity of bioactive compounds from Zingiberaceae plants and their synthetic derivatives has highlighted their potential as prospective antimicrobial agents. Nevertheless, the compounds from each included Zingiberaceae species should be tested on diverse groups of microorganisms, including resistant strains, to examine their potential antimicrobial efficacy. In fact, combining plant-derived compounds or their structurally modified analogues with commercially available antimicrobial agents could be a promising strategy to overcome the challenges elicited by resistant microbes. Moreover, more research should be carried out to elucidate the antimicrobial mechanisms of action, pharmacodynamics and pharmacokinetics of these compounds. Substantial *in vivo* studies and clinical trials are warranted to translate their roles into therapeutic practice in humans. The development of novel antimicrobial agents should consider the factors that may influence the efficacy of either bioactive constituents or synthetic analogues, such as tissue penetration, bioavailability and drug plasma levels.

## Figures and Tables

**Fig. (8):**
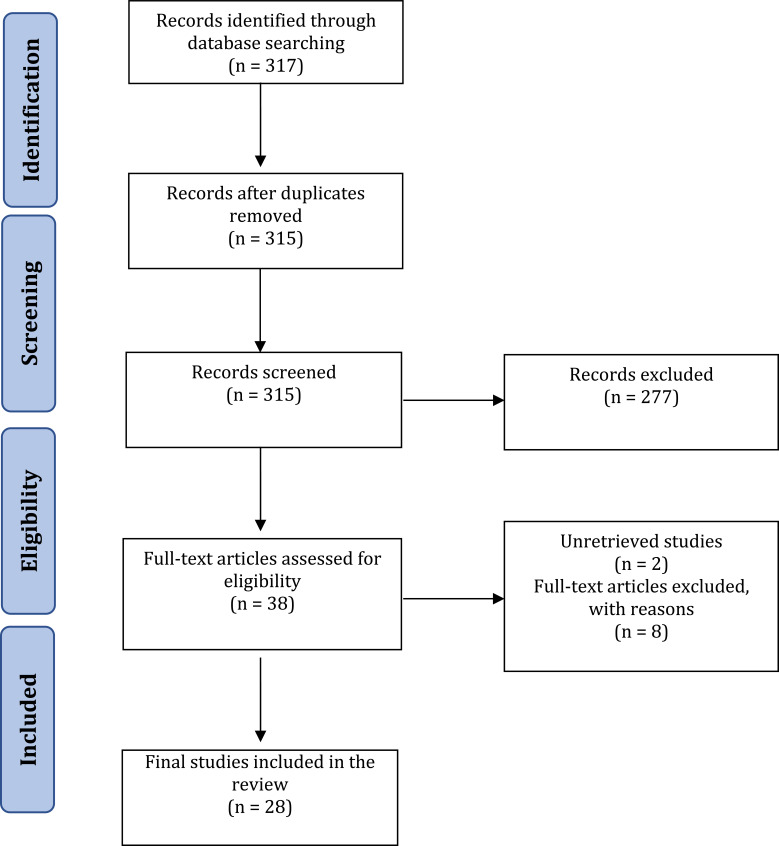
The preferred reporting items for systematic reviews and meta-analyses (PRISMA) extension for scoping reviews flow diagram.

**Fig. (9):**
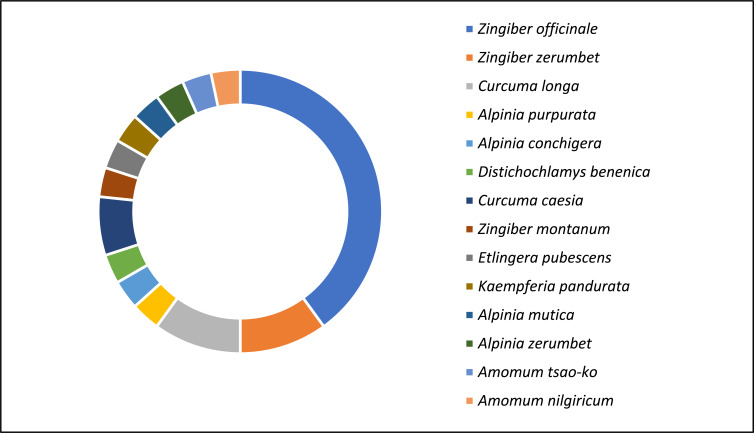
Species of the Zingiberaceae family involved in antimicrobial testing.

**Fig. (10):**
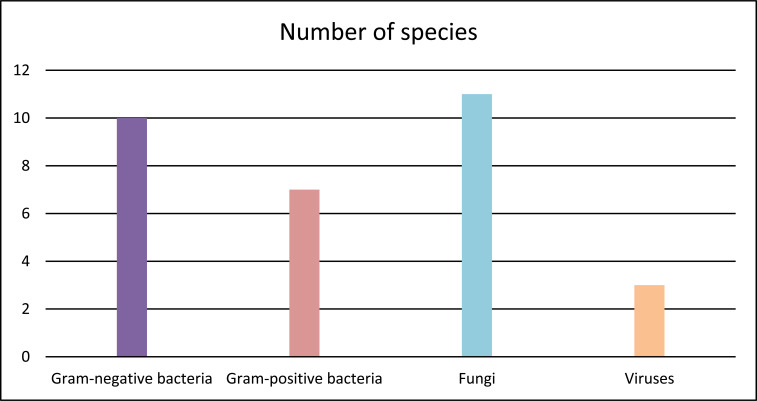
Microorganisms that were evaluated in the included studies.

**Table 1:** Keywords and search strings for literature search.

**Keywords**	**Search Strings**
*#*1 Bioactive compound	(‘Plant bioactive constituent$’ OR ‘Bioactive constituent$’ OR ‘Plant bioactive compound$’ OR ‘Bioactive compound$’ OR ‘Plan biologically active compound$’ OR ‘Biologically active compound$’ OR ‘Bioactive metabolite$’ OR ‘Phytochemical compound$’ OR ‘Phytonutrient$’ OR ‘Plant-derived compound$’ OR ‘Plant derived compound$’ OR ‘Plant-derived chemical$’ OR ‘Plant derived chemical$’ OR ‘Phenolic compound$’ OR Phenol$ OR Polyphenol$ OR Terpene$ OR Terpenoid$ OR Isoprenoid$)
#2 Zingiberaceae	(‘*Zingiber officinale* Roscoe’ OR ginger OR Zingiberaceae OR *Zingiber*)
#3 Antimicrobial	(Antibacterial OR Antimicrobial OR Antifungal OR Antiviral)
#4	#1 AND #2 AND #3

## Data Availability

The data and supportive information are available within the article.

## LIST OF ABBREVIATIONS

3CLpro: 3C-like Protease
AAK1: Adaptor-associated Kinase 1
ABC: ATP-binding Cassette
ACE2: Angiotensin-converting Enzyme 2
ADMET: Absorption, Distribution, Metabolism, Excretion and Toxicity
CPE: Cytopathic Effect
FIC: Fractional Inhibitory Concentration
IC_50_: Half-maximal Inhibitory Concentration
MBC: Minimum Bactericidal Concentration
MIC: Minimum Inhibitory Concentration
Mpro: Main Protease
MTT: 3-(4,5-dimethylthiazol-2-yl)-2,5-diphenyltetrazolium Bromide
NSP16: Non-structural Protein 16
PACs: Proanthocyanidins
PEDV: Porcine Epidemic Diarrhoea Virus
PL_pro_: Papain-like Protease
RBD: Receptor-binding Domain
RdRP: RNA-dependent RNA Polymerase
RMSD: Root Mean Square Deviation
RMSF: Root Mean Square Fluctuation

## STANDARDS OF REPORTING

PRISMA guidelines and methodology were followed.
