# Supplementary material for: Antimicrobial Potential of Natural Compounds of Zingiberaceae Plants and their Synthetic Analogues: A Scoping Review of In vitro and In silico Approaches
Source: Curr Top Med Chem. 2024 Apr 4;24(13):1158–84. doi: 10.2174/0115680266294573240328050629 (PMC11337242; doi:10.2174/0115680266294573240328050629)
Supplement: Supplementary file 1 [file CTMC-24-1158_SD1.pdf]

Supplementary Material

Antimicrobial Potential of Natural Compounds of Zingiberaceae Plants and their Synthetic Analogues: A Scoping Review of *in vitro* and *in silico* Approaches

Kok-Hou Yit[1] and Zamirah Zainal-Abidin[1,*]

[1]*Department of Craniofacial Diagnostics & Biosciences, Faculty of Dentistry, Universiti Kebangsaan Malaysia, 50300, Kuala Lumpur, Malaysia*

PRISMA 2020 flow diagram for new systematic reviews which included searches of databases and registers only

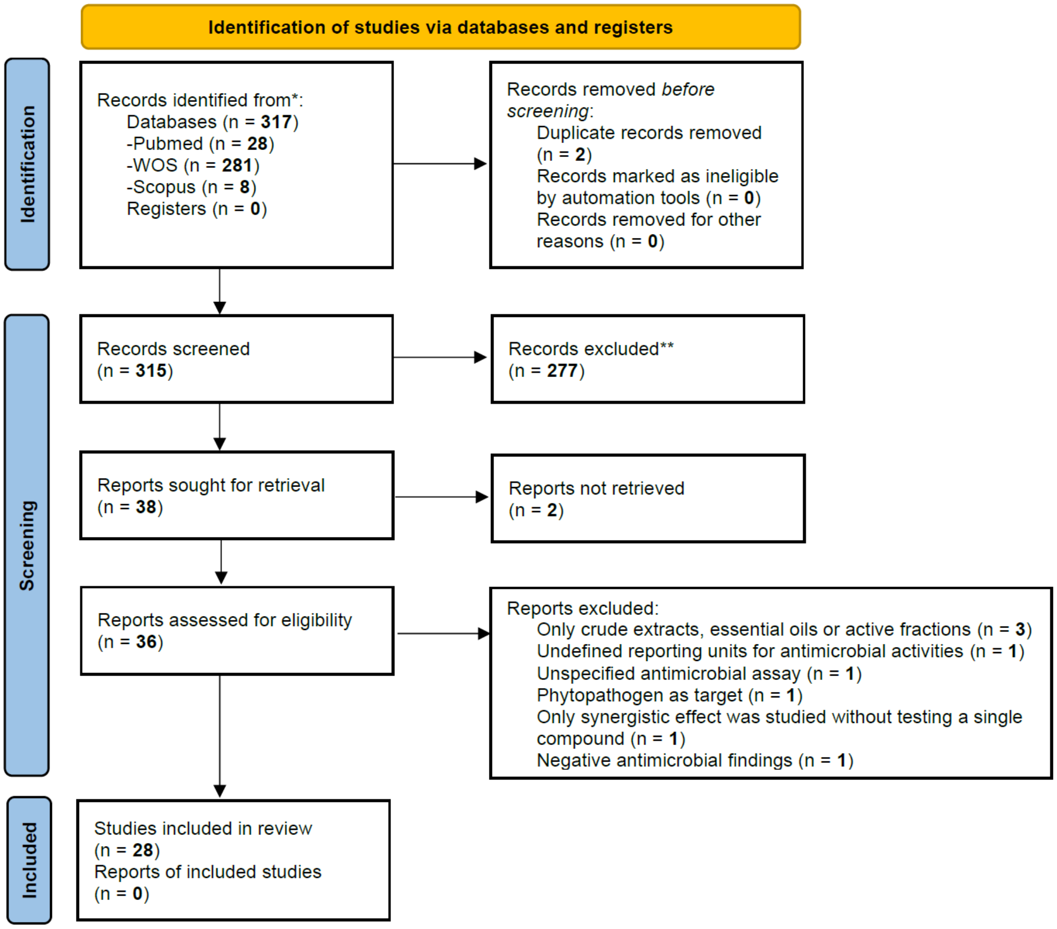

*Consider, if feasible to do so, reporting the number of records identified from each database or register searched (rather than the total number across all databases/registers).

**If automation tools were used, indicate how many records were excluded by a human and how many were excluded by automation tools.

From: Page MJ, McKenzie JE, Bossuyt PM, Boutron I, Hoffmann TC, Mulrow CD, et al. The PRISMA 2020 statement: an updated guideline for reporting systematic reviews. BMJ 2021;372:n71. doi: 10.1136/bmj.n71
For more information, visit: http://www.prisma-statement.org/

1873-4294/24 $65.00+.00 © 2024 Bentham Science Publishers

Table S1. Main results of antibacterial studies.

References	Type of Study	Zingiberaceae species	Compounds	Methodology	Bacterial species	Antibacterial activity			Study outcomes
						ZI (mm)	MIC	MBC	
[54]	*In vitro*	*Distichochlamys benenica* Q.B.Nguyen & Škorničk.	*Trans-o*-coumaric acid[1]<br>Borneol[2]<br>*Trans*-cinnamic acid[3]	Isolation: Silica-gel column chromatography<br>Characterisation: IR spectroscopy, ESI-MS, NMR<br>Assay: Broth microdilution	*Staphylococcus aureus*<br>*Bacillus subtilis*<br>*Pseudomonas aeruginosa*<br>*Escherichia coli*	NA	1.52 mM[1]<br>6.49 mM[2]<br>1.69 mM[3]<br>3.04 mM[1]<br>6.49 mM[2]<br>3.37 mM[3]<br>6.10 mM[1]<br>6.49 mM[2]<br>6.75 mM[3]<br>6.1 mM[1]<br>6.49 mM[2]<br>6.75 mM[3]	NA	Three of the compounds exhibited relatively low antibacterial activity against the tested gram-negative bacteria (*P. aeruginosa* and *E. coli*).
[47]	*In vitro*	*Kaempferia pandurate* Roxb.	Pinostrobin[4]<br>Monooxyprenylated chalcone[5]<br>Diprenylated chalcone[6]<br>Triprenylated cyclohexene chalcone[7]<br>Triprenylated chalcone[8]	Synthesis method:<br>Prenylation of pinostrobin<br>Characterisation: TLC, NMR, HRMS<br>Assay: Broth microdilution	*Bacillus subtilis*<br>*Staphylococcus aureus*<br>*Escherichia coli*	NA	37.5 µg/mL[4]<br>50 µg/mL[5]<br>50 µg/mL[6]<br>50 µg/mL[7]<br>50 µg/mL[8]<br>50 µg/mL[9]<br>150 µg/mL[4]<br>25 µg/mL[5]<br>25 µg/mL[6]<br>25 µg/mL[7]<br>25 µg/mL[8]<br>25 µg/mL[9]<br>37.5 µg/mL[4]<br>25 µg/mL[5]<br>25 µg/mL[6]<br>25 µg/mL[7]<br>25 µg/mL[8]<br>25 µg/mL[9]	NA	All the tested compound derivatives **(5–9)** had moderate or significant antibacterial activity. The lipophilicity of the compound derivatives could be enhanced by substituting prenyl groups on the flavone ring system, thus facilitating the interaction with the bacterial membranes.

			Monooxyprenyl-ated pinostrobin[9]		*Pseudomonas aeruginosa*		75 µg/mL[4]<br>25 µg/mL[5]<br>25 µg/mL[6]<br>25 µg/mL[7]<br>25 µg/mL[8]<br>25 µg/mL[9]		
[68]	*In vitro*	*Alpinia conchigera* Griff.	1′S-1′-Acetoxy-chavicol acetate[10]<br><br>*Trans-p*-coumaryl diacetate[11]	Isolation: Silica-gel column chromatography stepwise gradient system<br><br>Characterisation: NMR, IR spectroscopy, GC-EIMS<br><br>Assay: Broth microdilution	Methicillin-resistant *Staphylococcus aureus*	NA	0.5 mg/mL[10]<br><br>1.0 mg/mL[11]	NA	1′S-1′-acetoxy-chavicol acetate was the major component in *A. conchigera*, and it had better antibacterial activity than the other two isolated compounds.
[69]	*In vitro*	*Alpinia mutica* Roxb.	Auranamide[12]	Isolation: Vacuum column chromatography, recrystallisation<br><br>Characterisation: IR spectroscopy, MS, NMR<br><br>Assay: Broth microdilution	*Bacillus subtilis* ATCC 6633<br><br>*Staphylococcus aureus* ATCC 29737<br><br>*Escherichia coli* ATCC 10536<br>*Pseudomonas aeruginosa* ATCC 9027	NA	500 µg/mL[12]<br><br>500 µg/mL[12]<br><br>500 µg/mL[12]<br><br>500 µg/mL[12]	NA	Auranamide showed moderate inhibitory activity against all the tested bacteria.
[50]	*In vitro*	*Zingiber montanum* (J. Koenig) A.Dietr.	(*E*)-8(17),12-Labdadiene-15,16-dial[13]<br><br>Zerumbol[14]	Isolation: Vacuum liquid chromatography, solid phase extraction, column chromatography over Sephadex LH20, preparative thin layer chromatography	*Staphylococcus aureus* SA1199B<br><br>*Staphylococcus aureus* XU212<br><br>*S. aureus* ATCC 259 41<br><br>*S. aureus* RN4220	NA	0.212 mM[13]<br>0.291 mM[14]<br><br>0.424 mM[13]<br>0.582 mM[14]<br><br>0.212 mM[13]<br>0.582 mM[14]	NA	Compound 13 contains exo-methylene at C-8, an olefin at C-12, and two aldehyde groups at C-16 and 17. The presence of a hydroxyl group at C-1 in compound 14 could contribute to its significant antibacterial activity against the MRSA clinical isolates.

				Characterisation: NMR, HRMS<br><br>Assay:<br><br>Broth microdilution	Epidemic methicillin-resistant *S. aureus* 15<br><br>Methicillin-resistant *S. aureus* 27819<br><br>Methicillin-resistant *S. aureus* 340702		0.212 mM[13]<br>0.582 mM[14]<br><br>0.212 mM[13]<br>0.145–0.291 mM[14]<br><br>0.424 mM[13]<br>0.582 mM[14]<br><br>0.424 mM[13]<br>>0.582 mM[14]		
[49]	*In vitro*	*Zingiber zerumbet* (L.) Sm.	Zerumbol[14]<br><br>Zerumbone[15]<br><br>Zerumbone oxime[16]<br><br>Azazerumbone 1[17]<br><br>Azazerumbone 2[18]	Isolation: Silica-gel column chromatography<br><br>Characterisation: NMR, ESI-HRMS, IR spectroscopy, GC, TLC<br><br>Assay: Pour plate method	*Bacillus cereus*<br><br>*Staphylococcus aureus*<br><br>*Escherichia coli*<br><br>*Yersinia enterocolitica*	NA	60 ppm[14]<br>100 ppm[15]<br>60 ppm[16]<br>>1000 ppm[17]<br>75 ppm[18]<br><br>50 ppm[14]<br>125 ppm[15]<br>100 ppm[16]<br>>1000 ppm[17]<br>100 ppm[18]<br><br>75 ppm[14]<br>75 ppm[15]<br>100 ppm[16]<br>>1000ppm[17]<br>75 ppm[18]<br><br>125 ppm[14]<br>250 ppm[15]<br>150 ppm[16]<br>>1000 ppm[17]<br>125 ppm[18]	NA	The antibacterial activity of zerumbone synthetic derivatives could be due to the chemically modified carbonyl moiety in the zerumbone ring.
[53]	*In vitro*	*Zingiber zerumbet* (L.) Sm.	Zerumbone[15]	Synthesis method: Recrystallisation	*Enterococcus faecalis* ATCC 29212	At 100 mg/mL<br><br>10.00 ± 0.68[15]	250.00 µg/mL[15]	250.00 µg/mL[15]	Isolated zerumbone alone was more effective against all the target bacterial strains compared with the fresh and dried

				Characterisation: HPLC, EI-MS, NMR<br><br>Assay: Agar disc diffusion, Broth microdilution	*Staphylococcus aureus* ATCC 6538P<br><br>*Bacillus subtilis* CMCC (B) 63501<br><br>*Pseudomonas aeruginosa* CMCC (B) 10104<br>*Escherichia coli* ATCC 25922<br><br>*Proteus vulgaris* CMCC (B) 49027	15.72 ± 2.42[15]<br><br>9.48 ± 1.96 [15]<br><br>9.71 ± 0.97 [15]<br><br>10.85 ± 0.83[15]<br><br>11.71 ± 1.55[15]	31.25 µg/mL[15]<br><br>62.50 µg/mL[15]<br><br>250.00 µg/mL[15]<br><br>62.50 µg/mL[15]<br><br>62.50 µg/mL[15]	62.50 µg/mL[15]<br><br>125.00 µg/mL[15]<br><br>250.00 µg/mL[15]<br><br>62.50 µg/mL[15]<br><br>62.50 µg/mL[15]	rhizome essential oils.
[70]	*In vitro*	*Zingiber zerumbet* (L.) Sm.	Zerumbone[15]	Synthesis method: Hydro-distillation, recrystallisation<br><br>Characterisation: TLC, HPLC, LC-MS<br><br>Assay: Broth microdilution	*Helicobacter pylori*<br><br>NCTC11637<br><br>*H. pylori*<br><br>J99	NA	250 µg/mL[15]<br><br>250 µg/mL[15]	NA	Zerumbone exhibited significant antibacterial efficacy against *H. pylori*. This compound could be used as an alternative to treat peptic ulcers caused by *H. pylori* infection.
[45]	*In vitro*	*Zingiber officinale* Roscoe	Dehydrozingerone[19]<br><br>Dehydrozingerone 4-*O*-*β*-D-glucopyranoside tetra acetate[20]<br><br>4-*O*-*β*-D-Glucopyranosyl dehydrozingerone[21]<br><br>4-*O*-Acetyl dehydrozingerone[22]<br><br>Methyl ether of dehydrozingerone[23]	Characterisation: UV–Vis, FTIR, ES-MS, NMR<br><br>Assay: Agar well diffusion	*Bacillus subtilis*	At 2 µM<br>8.0[19]<br>13.0[20]<br>8.0[21]<br>11.5[22]<br>11.0[23]<br><br>At 5 µM<br>14.5[19]<br>19.0[20]<br>13.0[21]<br>15.0[22]<br>14.0[23]<br><br>At 10 µM<br>20.5[19]<br>24.5[20]<br>23.0[21]<br>20.0[22]<br>22.0[23]	NA	NA	The antibacterial activity of dehydrozingerone and its derivatives could be due to the presence of a conjugation system in the structural compounds, including an *α*,*β*-unsaturated carbonyl group.

						At 20 µM<br>29.0[19]<br>34.0[20]<br>30.0[21]<br>29.0[22]<br>30.5[23]			
					Listeria mono-cytogenes Scott-A	At 2 µM<br>9.0[19]<br>8.0[20]<br>8.0[21]<br>7.5[22]<br>11.0[23]<br>At 5 µM<br>14.0[19]<br>12.0[20]<br>11.0[21]<br>11.5[22]<br>14.5[23]<br>At 10 µM<br>21.5[19]<br>14.0[20]<br>16.5[21]<br>16.0[22]<br>21.5[23]<br>At 20 µM<br>29.0[19]<br>34.0[20]<br>30.0[21]<br>29.0[22]<br>23.5[23]			
					Escherichia coli MTCC 118	At 2 µM<br>9.0[19]<br>8.0[20]<br>8.0[21]<br>8.0[22]<br>9.0[23]<br>At 5 µM<br>13.5[19]<br>11.0[20]<br>12.0[21]<br>10.5[22]<br>13.5[23]<br>At 10 µM<br>18.0[19]<br>15.5[20]<br>13.5[21]<br>15.0[22]<br>17.0[23]<br>At 20 µM			

						21.0[19]<br>19.0[20]<br>15.5[21]<br>19.0[22]<br>22.0[23]<br><br>At 2 µM<br>8.0[19]<br>8.0[20]<br>9.5[21]<br>7.5[22]<br>9.0[23]<br><br>At 5 µM<br>12.0[19]<br>11.5[20]<br>15.0[21]<br>12.5[22]<br>14.0[23]<br><br>At 10 µM<br>15.0[19]<br>14.0[20]<br>15.0[21]<br>16.0[22]<br>21.0[23]<br><br>At 20 µM<br>18.5[19]<br>15.5[20]<br>18.5[21]<br>19.0[22]<br>26.5[23]			
					Salmonella enterica serotype Typhi				
[55]	*In vitro*	*Etlingera pubescens* (B.L.Burtt & R.M.Sm.) R.M.Sm.	Etlingerin[24]	Isolation: Sequential solvent extraction, C18 gravity column chromatography, preparative HPLC<br><br>Characterisation: NMR<br><br>Assay: Broth microdilution	*Staphylococcus aureus* ATCC 43300<br><br>*S. aureus* ATCC 6538P<br><br>*Bacillus cereus* ATCC 14579<br><br>*Bacillus subtilis* ATCC 8188	NA	0.125 mg/mL[24]<br><br>0.125 mg/mL[24]<br><br>0.0625 mg/mL[24]<br><br>0.0625 mg/mL[24]	0.125 mg/mL[24]<br><br>0.125 mg/mL[24]<br><br>0.0625 mg/mL[24]<br><br>0.0625 mg/mL[24]	Etlingerin could alter the bacterial membrane permeability, subsequently causing leakage of intracellular components in a dose-dependent manner (2× MIC > 1× MIC), thus suggesting its antibacterial mechanism.
[52]	*In vitro*	*Curcuma caesia* Roxb.	Curcumin[25]	Isolation: Silica-gel column chromatography	*Bacillus cereus*<br><br>*Bacillus subtilis*	NA	0.0625 mg/mL[25]<br><br>0.25 mg/mL[25]	0.0625 mg/mL[25]<br><br>0.25 mg/mL[25]	Purified curcumin exhibited greater antibacterial activity against the tested gram-positive

				Characterisation: HPLC,<br>UV-Vis spectroscopy, FTIR, NMR, and ESI-MS<br><br>Assay: Broth macrodilution,<br>spot inoculation	*Staphylococcus aureus*<br><br>*Staphylococcus epidermidis*<br><br>*Escherichia coli*<br><br>*Klebsiella pneumoniae*<br><br>*Pseudomonas aeruginosa*<br><br>*Proteus vulgaris*		0.25 mg/mL[25]<br><br>0.125 mg/mL[25]<br><br>0.50 mg/mL[25]<br><br>0.25 mg/mL[25]<br><br>1 mg/mL[25]<br><br>0.50 mg/mL[25]	0.25 mg/mL[25]<br><br>0.125 mg/mL[25]<br><br>0.50 mg/mL[25]<br><br>0.25 mg/mL[25]<br><br>1 mg/mL[25]<br><br>0.50 mg/mL[25]	and gram-negative bacteria compared with the crude extract.
[71]	*In vitro*	*Curcuma longa* L.	Monocurcuminoid CN 59[26]<br><br>Monocurcuminoid CN 63[27]<br><br>Monocurcuminoid CN 67[28]<br><br>Monocurcuminoid CN 77[29]<br><br>Curcumin[5]	Characterisation: FTIR, GC-MS, NMR<br><br>Assay: Broth microdilution	*Aeromonas hydrophila*<br><br>*Escherichia coli* ATCC 8733<br><br>*Enterococcus faecalis* ATCC 29212<br><br>*Klebsiella pneumoniae* ATCC 1705	NA	11.25 µg/mL[25]<br>-[26]<br>-[27]<br>3.75 µg/mL[28]<br>35 µg/mL[29]<br><br>11.25 µg/mL[25]<br>130 µg/mL[26]<br>4.69 µg/mL[27]<br>30 µg/mL[28]<br>130 µg/mL[29]<br><br>22.5 µg/mL[25]<br>130 µg/mL[26]<br>75 µg/mL[27]<br>30 µg/mL[28]<br>35 µg/mL[29]<br><br>90 µg/mL[25]<br>65 µg/mL[26]<br>18.75 µg/mL[27]	45 µg/mL[25]<br>-[26]<br>-[27]<br>15 µg/mL[28]<br>140 µg/mL[29]<br><br>45 µg/mL[25]<br>-[26]<br>18.75 µg/mL[27]<br>120 µg/mL[28]<br>-[29]<br><br>90 µg/mL[25]<br>-[26]<br>300 µg/mL[27]<br>120 µg/mL[28]<br>140 µg/mL[29]<br><br>360 µg/mL[25]<br>260 µg/mL[26]	CN 59 displayed very strong antibacterial activity against *P. aeruginosa*, CN 63 possessed very strong antibacterial activity against *E. coli*, while CN 67 exhibited a very strong antibacterial effect only against *A. hydrophila*.

					Pseudomonas aeruginosa ATCC 27853<br><br>*Staphylococcus aureus* ATCC 6538		120 µg/mL[28]<br>140 µg/mL[29]<br><br>45 µg/mL[25]<br>4.06 µg/mL[26]<br>75 µg/mL[27]<br>30 µg/mL[28]<br>17.5 µg/mL[29]<br><br>45 µg/mL[25]<br>16.25 µg/mL[26]<br>150 µg/mL[27]<br>120 µg/mL[28]<br>35 µg/mL[29]	75 µg/mL[27]<br>-[28]<br>-[29]<br><br>180 µg/mL[25]<br>16.25 µg/mL[26]<br>300 µg/mL[27]<br>120 µg/mL[28]<br>70 µg/mL[29]<br><br>180 µg/mL[25]<br>65 µg/mL[26]<br>150 µg/mL[27]<br>120 µg/mL[28]<br>140 µg/mL[29]	
[48]	*In vitro*	*Zingiber officinale* Roscoe	6-Gingerol[30]<br><br>6-Shogaol[31]	Characterisation: TLC<br><br>Assay: Broth microdilution	*Staphylococcus aureus* SA1199B<br><br>*S. aureus* ATCC 25923<br><br>*Bacillus subtilis* BsSOP01<br><br>Methicillin-resistant *S. aureus* XU212<br><br>Epidemic methicillin-resistant *S. aureus* 15<br><br>Epidemic methicillin-resistant *S. aureus* 16<br><br>*S. aureus* RN 4220	NA	16 mg/L[30]<br>16 mg/L[31]<br><br>128 mg/L[30]<br>128 mg/L[31]<br><br>64 mg/L[30]<br>NT[31]<br><br>16 mg/L[30]<br>16 mg/L[31]<br><br>64 mg/L[30]<br>32 mg/L[31]<br><br>512 mg/L[30]<br>512 mg/L[31]	NA	6-Shogaol had better antibacterial activity against gram-negative bacteria than 6-gingerol, despite the structural barrier of the bacteria.

					Methicillin-resistant *S. aureus* 346724<br><br>Methicillin-resistant *S. aureus* 774812<br><br>Methicillin-resistant *S. aureus* 274829<br><br>Methicillin-resistant *S. aureus* 12981<br><br>*Enterococcus faecalis* 13379<br><br>*Escherichia coli* NCTC 10418<br><br>*Pseudomonas aeruginosa* 10662<br><br>*Klebsiella pneumoniae* 342<br><br>*Proteus* sp. 10830		128 mg/L[30]<br>8 mg/L[31]<br><br>128 mg/L[30]<br>16 mg/L[31]<br><br>512 mg/L[30]<br>512 mg/L[31]<br><br>64 mg/L[30]<br>64 mg/L[31]<br><br>512 mg/L[30]<br>128 mg/L[31]<br><br>8 mg/L[30]<br>NT[31]<br><br>512 mg/L[30]<br>128 mg/L[31]<br><br>512 mg/L[30]<br>128 mg/L[31]<br><br>512 mg/L[30]<br>128 mg/L[31]<br><br>512 mg/L[30]		

							512 mg/L[31]		
[46]	*In vitro*	*Curcuma longa* L.[1]<br><br>*Zingiber officinale* Roscoe[2]	Tetrahydrocurcumin[33]<br><br>1-(2-(4-Hydroxy-3-methoxy-phenethyl)-4-methylquinolin-3-yl)-3-(4-hydroxy-3-methoxy-phenyl)propan-1-one[34]<br><br>1-(2-(4-Hydroxy-3-methoxy-phenethyl)-4-phenylquinolin-3-yl)-3-<br>(4-hydroxy-3-methoxy-phenyl)propan-1-one[35]<br><br>1-(2-(4-Hydroxy-3-methoxy-phenethyl)-6-nitro-4-phenylquinolin3-yl)-3-(4-hydroxy-3-methoxy-phenyl)propan-1-one[36]<br><br>1-(6-Chloro-2-(4-hydroxy-3-methoxyphenethyl)-4-phenylquinolin-3-yl)-3-(4-hydroxy-3-methoxy-phenyl)propan-1-one[37]<br><br>1-(6-Amino-2-(4-hydroxy-3-methoxyphenethyl)-4-phenylquinolin-3-yl)-3-(4-hydroxy-	Synthesis method: Trituration of crude product<br><br>Characterisation: TLC, NMR, ESI-MS, UV-Vis<br><br>Assay: Pour plate method	*Bacillus cereus* F 4810<br><br>*Staphylococcus aureus* FRI 722<br><br>*Yersinia enterocolitica* MTCC 851<br><br>*Escherichia coli* MTCC 108	NA	1049 µM[33]<br>724 µM[34]<br>503 µM[35]<br>422 µM[36]<br>774 µM[37]<br>534 µM[38]<br>649 µM[39]<br>1256 µM[32]<br>>2000 µM[40]<br>412 µM[41]<br>609 µM[42]<br>219 µM[43]<br>593µM[44]<br><br>1311 µM[33]<br>983 µM[34]<br>914 µM[35]<br>674 µM[36]<br>946 µM[37]<br>800 µM[38]<br>973 µM[39]<br>>2000 µM[32]<br>>2000 µM[40]<br>1098µM[41]<br>792 µM[42]<br>501 µM[43]<br>790 µM[44]<br><br>2098 µM[33]<br>931 µM[34]<br>686 µM[35]<br>506 µM[36]<br>860 µM[37]<br>711 µM[38]<br>811 µM[39]<br>>2000 µM[32]<br>>2000 µM[40]<br>1030 µM[41]<br>853 µM[42]<br>752 µM[43]<br>856 µM[44]<br><br>1704 µM[33]<br>1242 µM[34]<br>823 µM[35]<br>843 µM[36]<br>817 µM[37]<br>978 µM[38]<br>933 µM[39]<br>>2000 µM[32]<br>>2000 µM[40]<br>1236 µM[41]<br>975 µM[42]	NA	Compounds 43 and 36 were the most effective against gram-negative and gram-positive bacteria, respectively. Modification of the carbonyl group in the side chain of tetrahydrocurcumin and zingerone could improve the antibacterial activity.

			3-methoxy-phenyl)propan-1-one[38]<br><br>1-(6-Chloro-4-(2-chlorophenyl)-2-(4-hydroxy-3-methoxy-phenethyl) quino-lin-3-yl)-3-(4-hy-droxy-3-methoxy-phenyl)propan-1-one[39]<br><br>Zingerone[32]<br><br>4-((2,4-Dime-thylquinolin-3-yl)methyl)-2-methoxyphenol[40]<br><br>2-Methoxy-4-((2-methyl-4-phe-nylquinolin-3-yl)methyl)phe-nol[41]<br><br>2-Methoxy-4-((2-methyl-6-nitro-4-phenylquinolin-3-yl)methyl)phe-nol[42]<br><br>4-((6-Chloro-2-methyl-4-phe-nylquinolin-3-yl)methyl)-2-methoxyphenol[43]<br><br>4-((6-Amino-2-methyl-4-phe-nylquinolin-3-yl)methyl)-2-methoxyphenol[44]				877 µM[43]<br>1054 µM[44]		

[56]	*In vitro*<br><br>*In silico*	*Zingiber officinale* Roscoe	Lariciresinol[45]	Isolation: Bioassay-guided fractionation<br><br>Characterisation: TLC<br><br>Assay: Broth microdilution, molecular docking	*Salmonella enterica* serovar *typhimurium* NKS773<br><br>*S. enterica* serovar *typhimurium* NKS70<br><br>*S. enterica* serovar *typhimurium* NKS174	NA	400 µg/mL[45]<br><br>400 µg/mL[45]<br><br>400 µg/mL[45]	NA	Lariciresinol showed good affinity to RamR and RamA, with docking scores of -8.2 and -7.4 kcal/mol, respectively. This compound also decreased the MIC of tetracycline by 2–4-fold, suggesting its synergistic or additive effects.
[51]	*In vitro*	*Amomum tsaoko* Crevost & Lemarié	Tsaokol B[46]<br><br>(*E*)-2-Decene-1,10-diol[47]<br><br>Tsaokol A[48]<br><br>Acetoxytsaokol A[49]<br><br>(2*E*,8*E*)-2,8-Decadiene-1,10-diol[50]<br><br>(2*E*,6*E*)-1,8-Diacetoxy-2,6-octadiene[51]<br><br>(*E*)-Decenal[52]<br><br>(*E*)-Dec 2-enyl acetate[53]	Isolation: Silica gel, Sephadex LH-20 and Diaion HP-20 column chromatography, prepacked cartridges, Redi Sep-Silica and Redi Sep-C18 flash chromatography, preparative HPLC<br><br>Characterisation: TLC, NMR, UV-Vis, FT-IR, HR-DART-MS<br><br>Assay: Microplate Alamar blue assay (for	*Mycobacterium tuberculosis* H37Rv (replicating)	NA	10.2 µg/mL[46]<br>29.7 µg/mL[47]<br>0.6 µg/mL[48]<br>1.1 µg/mL[49]<br>4.3 µg/mL[50]<br>26.1 µg/mL[51]<br>>100 µg/mL[52]<br>>100 µg/mL[53]<br>>100 µg/mL[54]<br>>100 µg/mL[55]<br>88.6 µg/mL[56]<br>>100	NA	Tsaokol A had the greatest inhibitory effect against replicating and non-replicating *M. tuberculosis* H37Rv. The MIC ratio of the non-replicating to replicating strain for tsaokol A was comparable to the first-line tuberculosis drugs isoniazid and rifampin. The antibacterial activity could be attributed to the olefinic group at the C-8 position.

			(*E*)-2-Dodecen-1-yl acetate[54]<br><br>Geraniol[55]<br><br>Geranyl acetate[56]<br><br>(3*R*)-(*E*)-Nerolidol[57]	replicating strain), low oxygen recovery assay (for non-replicating strain)	*M. tuberculosis* H37Rv (non-replicating)		µg/mL[57]<br><br>61.5 µg/mL[46]<br>95.4 µg/mL[47]<br>1.4 µg/mL[48]<br>21.6 µg/mL[49]<br>5.9 µg/mL[50]<br>>100 µg/mL[51]<br>>100 µg/mL[52]<br>>100 µg/mL[53]<br>>100 µg/mL[54]<br>>100 µg/mL[55]<br>>100 µg/mL[56]<br>>100 µg/mL[57]		
[72]	*In silico*	*Amomum nilgiricum* V.P.Thomas & M.Sabu	Serverogenin acetate[58]	Characterisation: GC-MS<br><br>Assay: Molecular docking	NA	NA	NA	NA	The docking results revealed a binding energy of -4.22 kcal/mol. There was a hydrogen bond between O26 in serverogenin acetate and Gly341 in the 5iwm bacterial target protein.
[73]	*In vitro*<br><br><br>*In silico*	*Curcuma caesia* Roxb.	Muurola-4–10(14)-dien-1-ol[59]<br><br>Globulol[60]<br><br>Viridiflorol[61]<br><br>Germacrone[62]<br><br>Epicurzerenone[63]<br><br>Curzerenone[64]<br><br>Eucalyptol[65]<br><br>Camphor[66]	Characterisation: GC-MS<br><br>Assay: Broth microdilution, agar well diffusion, molecular docking	Multidrug-resistant *Escherichia coli*<br><br><br><br><br><br><br><br>Multidrug-resistant *Staphylococcus aureus*	3.02 ± 0.02[59]<br>8 ± 0.02[60]<br>9.06 ± 0.02[61]<br>6.03 ± 0.02[62]<br>7 ± 0.02[63]<br>10 ± 0.02[64]<br>8.02 ± 0.02[65]<br>8 ± 0.04[66]<br><br><br><br>4.01 ± 0.01[59]<br>9.01 ± 0.02[60]<br>10.25 ± 0.02[61]<br>7.5 ± 0.02[62]<br>8.02 ± 0.01[63]	25 ± 0.02[59] 12.5 ± 0.01[60]<br>12.5 ± 0.02[61] 25 ± 0.02[62] 6.25 ± 0.02[63]<br>3.125 ± 0.02[64]<br>12.5 ± 0.02[65] 12.5 ± 0.02[66]<br><br><br>12.5 ± 0.02[59] 6.25 ± 0.02[60]<br>3.12 ± 0.02[61] 12.5 ± 0.02[62]<br>3.12 ± 0.02[63] 1.56 ± 0.01[64]<br>6.25 ± 0.01[65] 6.25 ± 0.01[66]	25 ± 0.02[59] 12.5 ± 0.02[60]<br>12.5 ± 0.02[61] 25 ± 0.02[62] 12.5 ± 0.02[63]<br>6.25 ± 0.02[64] 12.5 ± 0.02[65]<br>12.5 ± 0.01[66]<br><br><br><br>12.5 ± 0.02[59] 6.25 ± 0.02[60]<br>6.25 ± 0.02[61] 12.5 ± 0.02[62]<br>3.12 ± 0.02[63] 3.12 ± 0.01[64]<br>6.25 ±	Among the tested compounds, viridiflorol demonstrated the best antibacterial activity against multidrug-resistant *E. coli* and *S. aureus* by producing the largest inhibition zones and the lowest MICs. *In silico* molecular docking revealed the lowest binding energy for viridiflorol (-9.02 kcal/mol) against *S. aureus* tyrosyl-tRNA synthetase by hydrogen bonds with AspA40 and TyrA170. In contrast,

						11 ± 0.02[64]<br>9 ± 0.02[65]<br>8.8 ± 0.01[66]		0.01[65] 12.5 ± 0.01[66]	muurola-4–10(14)-dien-1-ol showed the best interaction with *E. coli* glucosamine 6-phosphate synthase at SerA174 (-6.09 kcal/mol).

Note: MIC = minimum inhibitory concentration; MBC = minimum bactericidal concentration; ZI = zone of inhibition; NT = not tested; NA = not available; IR = infrared; ESI-MS = electrospray ionization mass spectrometry; NMR = nuclear magnetic resonance; TLC = thin-layer chromatography; HRMS = high-resolution mass spectrometry; GC-EIMS = gas chromatography and electron ionization mass spectrometry; MS = mass spectrometry; ESI-HRMS = electrospray ionization high-resolution mass spectrometry; GC = gas chromatography; HPLC = high-performance liquid chromatography; EI-MS = electron ionization mass spectrometry; LC-MS = liquid chromatography-mass spectrometry; UV-Vis = ultraviolet-visible; FTIR = fourier transform infrared spectroscopy; ES-MS = Electrospray mass spectrometry

'-' indicates value was not within the tested concentration range.

The superscript numbers reported in the Supplementary Tables correspond to the bold numbers in the main text for each compound.

Table S2. Main results of antifungal studies.

**References**	**Type of study**	**Zingiberaceae species**	**Compounds**	**Methodology**	**Fungal species**	**Antifungal activity**			**Study outcomes**
						**ZI (mm)**	**MIC**	**MFC**	
[45]	*In vitro*	*Zingiber officinale* Roscoe	Dehydrozingerone[19]<br><br>Dehydrozingerone 4-*O*-*β*-D-glucopyranoside tetra acetate[20]<br><br>4-*O*-*β*-D-Glucopyranosyl dehydrozingerone[21]<br><br>4-*O*-Acetyl dehydrozingerone[22]<br><br>Methyl ether of dehydrozingerone[23]	Characterisation: UV-Vis, FTIR, ES-MS, NMR<br><br>Assay: Agar well diffusion	*Aspergillus niger*<br><br>*Aspergillus flavus*	at 1 mg<br><br>31.0 ± 1.0[19]<br>NI[20]<br>NI[21]<br>21.0 ± 1.04[22]<br>19.0 ± 1.05[23]<br><br>20.5 ± 2.5[19]<br>NI[20]<br>NI[21]<br>15.0 ± 1.0[22]<br>16.0 ± 2.0[23]<br><br>25.0 ± 5.0[19]<br>16.5 ± 1.5[20]<br>30.0 ±	NA	NA	The antifungal activity of dehydrozingerone and its derivatives may be attributed to the *α*,*β*-unsaturated carbonyl groups in their structures.

					Aspergillus oryzae<br><br>*Aspergillus ochraceus*<br><br>*Penicillium* spp.<br><br>*Fusarium sp.*	0.2[21]<br>25.0 ± 0.4[22]<br>24.5 ± 0.5[23]<br><br>28.5 ± 1.5[19]<br>25.0 ± 4.0[20]<br>16.5 ± 3.5[21]<br>27.5 ± 2.5[22]<br>25.0 ± 0.4[23]<br><br>31.5 ± 3.5[19]<br>21.0 ± 1.0[20]<br>17.5 ± 0.5[21]<br>30.5 ± 0.5[22]<br>26.5 ± 0.5[23]<br><br>18.5 ± 3.5[19]<br>19.5 ± 0.5[20]<br>19.0 ± 1.0[21]<br>24.0 ± 1.0[22]<br>21.0 ± 1.0[23]			
[72]	*In silico*	*Amomum nilgiricum* V.P.Thomas & M.Sabu	Serverogenin acetate[58]	Characterisation: GC-MS<br><br>Assay: Molecular docking	NA	NA	NA	NA	The docking results revealed a binding energy of -4.09 kcal/mol. Hydrogen bonds were formed between serverogenin acetate and Thr170, Arg173, Ser504, Lys503, His502 and His469 in the 4i9p fungal target protein.

[57]	*In vitro*	*Zingiber officinale* Roscoe	6-Shogaol[31]	Assay: Broth microdilution	*Candida auris* NCCP32683<br><br>*C. auris* NCCP32640<br><br>*C. auris* NCCP32641<br><br>*C. auris* NCCP32684<br><br>*C. auris* NCCP32685<br><br>*Candida glabrata* KBN12P0509	NA	50% inhibition: 16 μg/mL[56]<br>80% inhibition: 32 μg/mL[56]<br><br>50% inhibition: 16 μg/mL[56]<br>80% inhibition: 32 μg/mL[56]<br><br>50% inhibition: 16 μg/mL[56]<br>80% inhibition: 32 μg/mL[56]<br><br>50% inhibition: 16 μg/mL[56]<br>80% inhibition: 32 μg/mL[56]<br><br>50% inhibition: 32 μg/mL[56]<br>80% inhibition: 64 μg/mL[56]<br><br>50% inhibition: >64 μg/mL[56]	NA	6-Shogaol inhibited *C. auris* planktonic cells and biofilms in a dose-dependent manner. 6-Shogaol exerted a more effective antifungal effect on *C. auris* than fluconazole over time in the time-kill assay.

							80% inhibition: >64 µg/mL[56]		
					Candida tropicalis KBN12P0509		50% inhibition: 32 µg/mL[56]<br>80% inhibition: 64 µg/mL[56]		
					C. tropicalis KBN12P05100		50% inhibition: 64 µg/mL[56]<br>80% inhibition: >64 µg/mL[56]		
[53]	*In vitro*	*Zingiber zerumbet* (L.) Sm.	Zerumbone[15]	Synthesis method: Recrystallisation method<br>Characterisation: HPLC, EI-MS, NMR<br>Assay:<br>Agar disc diffusion, broth microdilution	*Candida albicans* CMCC (F) 9800	at 100 mg/mL<br>11.31 ± 0.83[15]	31.25 µg/mL[15]	250.00 µg/mL[15]	Isolated zerumbone alone was more effective against the tested fungus compared with the fresh and dried rhizome essential oils.
[71]	*In vitro*	*Curcuma longa* L.	Curcumin[25]<br>Monocurcuminoid CN 59[26]<br>Monocurcuminoid CN 63[27]	Characterisation: FTIR, GC-MS, NMR<br>Assay:<br>Broth microdilution	*Candida albicans* ATCC 24433	NA	22.5 µg/mL[25]<br>4.06 µg/mL[26]<br>4.69 µg/mL[27]<br>60 µg/mL[28]<br>140 µg/mL[29]	90 µg/mL[25]<br>16.25 µg/mL[26]<br>18.75 µg/mL[27]<br>240 µg/mL[28]<br>-[29]	Both CN 63 and CN 59 exhibited very strong antifungal activity against *C. albicans*, whereas curcumin only had strong activity.

			Monocurcuminoid CN 67[28]<br><br>Monocurcuminoid CN 77[29]						
[58]	*In vitro*	*Zingiber officinale* Roscoe	Dehydrozingerone[19]	Assay: Broth dilution	*Candida albicans*<br><br>*Saccharomyces cerevisiae*	NA<br><br>NA	2 mmol/L<br><br>2 mmol/L	NA<br><br>NA	When combined, dehydrozingerone could enhance the antifungal effect of glabridin against *C. albicans* and *S. cerevisiae* (based on the checkerboard assay). Besides, the combined treatment significantly downregulated the expression of the *PDR1*, *PDR3* and *PDR5* genes, which encode drug efflux pumps, compared with glabridin alone. Dehydrozingerone indirectly altered translation of GFP-tagged Pdr5p, an ABC transporter.
[73]	*In vitro*<br><br>*In silico*	*Curcuma caesia* Roxb.	Muurola-4–10(14)-dien-1-ol[59]<br><br>Globulol[60]<br><br>Viridiflorol[61]<br><br>Germacrone[62]<br><br>Epicurzerenone[63]<br><br>Curzerenone[64]<br><br>Eucalyptol[65]<br><br>Camphor[66]	Characterisation: GC-MS<br><br>Assay: Broth microdilution, Agar well diffusion, Molecular docking	*Candida albicans*	2 ± 0.01[59]<br>7.5 ± 0.01[60]<br>8.8 ± 0.02[61]<br>5 ± 0.02[62]<br>7 ± 0.02[63]<br>10.21 ± 0.02[64]<br>7 ± 0.02[65]<br>6 ± 0.01[66]	25 ± 0.02[59]<br>12.5 ± 0.01[60]<br>25 ± 0.01[61] 25 ± 0.01[62]<br>12.5 ± 0.02[63]<br>25 ± 0.02[64]<br>25 ± 0.04[65]<br>25 ± 0.015[66]	25 ± 0.02[59]<br>25 ± 0.01[60]<br>25 ± 0.01[61]<br>25 ± 0.01[62]<br>25 ± 0.01[63]<br>25 ± 0.02[64]<br>25 ± 0.04[65]<br>25 ± 0.015[66]	Globulol and epicurzerenone had the lowest MICs, while curzerenone produced the largest inhibition zone against *C. albicans*. Molecular docking supported the antifungal effect of globulol with maximum biding affinity (-8.43 kcal/mol), showing the formation of a stable complex with *N*-myristoyl transferase from *C. albicans* via a hydrogen bond at LeuA355.
[74]	*In silico*	*Alpinia purpurata*<br><br>(Vieill.) K.Schum.	Stigmasta-5,22-dien-3-ol, acetate, (3.beta.)[67]<br><br>Farnesyl acetate[68]	Assay: Molecular docking	*Candida krusei*	NA	NA	NA	Stigmasta-5,22-dien-3-ol, acetate, (3.beta.) could bind to His377 and Phe380 of the *C. krusei* target lanos-

									terol-14-α-demethylase with a binding energy of -13.53 kcal/mol, while farnesyl acetate interacted with His377 and Phe380 with a binding energy of -7.15 kcal/mol. Both docked compounds complied with Lipinski's rule of five.

Note: MIC = minimum inhibitory concentration; MFC = minimum fungicidal concentration; ZI = zone of inhibition; NA = not available; NI = no inhibition; UV-Vis = ultraviolet-visible; FTIR = fourier transform infrared spectroscopy; ES-MS = electrospray mass spectrometry; NMR = nuclear magnetic resonance; GC-MS = gas chromatography-mass spectrometry; HPLC = high-performance liquid chromatography; EI-MS = electron ionization mass spectrometry

'-' indicates value was not within the tested concentration range.

The superscript numbers reported in the Supplementary Tables correspond to the bold numbers in the main text for each compound.

Table (S3) Main results of antiviral studies.

**References**	**Type of study**	**Zingiberaceae species**	**Compounds**	**Methodology**	**Viral species**	**Antiviral activity**	**Study outcomes**
[60]	*In vitro*<br><br>*In silico*	*Zingiber officinale* Roscoe	24-Methylcholesta-7-en-3β-on[69]<br><br>Spinasterol[70]<br><br>Spinasterone[71]	Isolation: Vacuum column chromatography, preparative TLC<br><br>Characterisation: GC-MS, LC-MS/MS, NMR<br><br>Assay: SARS-CoV-2 3CL protease *in vitro* inhibition assay, molecular docking	SARS-CoV-2	24-Methylcholesta-7-en-3β-on, spinasterone and spinasterol displayed a binding energy of -68.80, -87.41 and -78.11 kcal/mol, respectively, to 3C-like protease, comparably lower than the positive controls baicalein (-47.14 kcal/mol) and remdesivir (-68.55 kcal/mol). For the *in vitro* study, 24-methylcholesta-7-en-3β-on at 200 µg/mL exhibited 75% inhibition of SARS-CoV-2 3C-like protease.	Three of these steroid compounds were more potent in inhibiting SARS-CoV-2 3C-like protease compared with other isolated compounds.
[66]	*In silico*	*Zingiber officinale* Roscoe	4-Gingerol[72]	Assay: Molecular docking	SARS-CoV-2	4-Gingerol displayed a binding energy of -7.3 kcal/mol with the SARS-CoV-2 main	4-Gingerol displayed the lowest binding energy. Thus, this compound could be

						protease. Moreover, 4-gingerol formed hydrophobic bonds with Phe294, Asn151, Gln110 and Val104, and hydrogen bonds with Asn151, Thr111 and Asp153 with the M^{pro} domain.	a candidate for drug design and development for the treatment of COVID-19 by inhibiting M^{pro} associated with viral inactivation and consequent failure of virion assembly.
[59]	*In silico*	*Zingiber officinale* Roscoe	Monoterpene[73]	Assay: Molecular docking	SARS-CoV-2	Monoterpene interacted with RNA-dependent RNA polymerase (RdRP) by forming a hydrogen bond with Ser15 and creating an alkyl bond with Lys411 and Met87; the binding energy was -4.7 kcal/mol. The compound formed a hydrogen bond with Gly110 and an alkyl bond with Lys152 in 3C-like protease ($3CL^{pro}$); the binding energy was -6.4 kcal/mol. Furthermore, monoterpene showed alkyl bonding with Lys103 and Ile106 in angiotensin-converting enzyme 2 (ACE2); the binding energy was -5.6 kcal/mol.	Monoterpene showed the best interaction with target proteins RdRP, $3CL^{pro}$, and ACE2, as suggested by the researchers.
[67]	*In silico*	*Zingiber officinale* Roscoe<br><br>*Curcuma longa* L.	Geraniin[74]<br><br>*O*-Demethyldemethoxy-curcumin[75]	Assay: Molecular docking	SARS-CoV-2	Geraniin and *O*-demethyl-demethoxycurcumin had a binding energy of 8.2 and 8 kcal/mol with the receptor binding domain (RBD) of the spike protein, respectively. Geraniin interacted hydrophobically with Leu368 and formed hydrogen bonds with Val362, Asp364, Val367 and Ser371 in the RBD of the spike protein. There were hydrogen bonds between *O*-demethyl-demethoxycurcumin and Cys336 and Asp364, and hydrophobic interactions between this compound and Leu335, Phe338, Asp364, Val367, Leu368 and Phe374 in the RBD of the spike protein.	Geraniin from *Z. officinale* and *O*-demethyl-demethoxy-curcumin from *C. longa* showed the highest binding affinity among the compounds found in each Zingiberaceae species.
[62]	*In silico*	*Zingiber officinale* Roscoe	3-[(2S,3R,4S,5S,6R)_4,5-Dihydroxy-6-(hydroxymethyl)_3-[(2S,3R,4S,5R)_3,	Assay: Molecular docking	SARS-CoV-2	Compounds 77 and 25 demonstrated strong binding affinity with 6VW1/6VSB from the spike protein, with a binding energy of -8.643/-	Compounds 77 and 25 could be potential SARS-CoV-2 viral entry inhibitors.

			4,5-rihydroxy-oxan-2-yl]oxy-oxan-2-yl]oxy-2-(3,4-dihydroxy-phenyl)_5-hy-droxy-7-methoxy-chromen-4-one[76]<br><br>Sissostrin[77]<br><br>Curcumin[25]			9.178 and -7.013/-8.216 kcal/mol, respectively. They could function as potential viral entry inhibitors. Besides compounds 77 and 25, compound 76 exhibited high binding affinity to 6LU7 and 6M03 of the SARS-CoV-2 main protease.	Moreover, compounds 76 and 77 could deregulate SARS-CoV-2 viral gene expression during replication.
[61]	*In silico*	*Zingiber officinale* Roscoe	Quercetin[78]<br><br>5,6-Epoxy cholestan-3-ol[79]<br><br>Riboflavin[80]	Characterisation: LC-MS<br><br>Assay: Molecular docking	SARS-CoV-2	Binding energy (kcal/mol) with adaptor-associated protein kinase 1 (AAK1):<br><br>-9[78]<br><br>-8.4[79]<br><br>-8.8[80]<br><br>Binding energy (kcal/mol) with cathepsin L:<br><br>-6.7[78]<br><br>-6.5[79]<br><br>-6.8[80]<br><br>Binding energy (kcal/mol) with the main protease ($M^{pro)}$:<br><br>-7.7[78]<br><br>-6.8[79]<br><br>-8[80]<br><br>Binding energy (kcal/mol) with ADP ribose phosphatase:<br><br>-8.4[78]<br><br>-8[79]<br><br>-7.4[80]<br><br>Binding energy (kcal/mol) with nonstructural protein 14 (NSP 14):<br><br>-8.9[78]	Quercetin, but not the other two compounds, interacted with Phe6947 and Gly6911. All three compounds interacted with Asp6912, Asp6897, Gly6869, Glu6871, Leu6898, Tyr6930, Cys6913 and Met6929.

						-9.2[79]<br><br>-9.5[80]<br><br>Binding energy (kcal/mol) with nonstructural protein 16 (NSP 16):<br><br>-8.2[78]<br><br>-7.5[79]<br><br>-7.0[80]<br><br>Binding energy (kcal/mol) with papain-like protease (PL^{pro}):<br><br>-7.7[78]<br><br>-7.3[79]<br><br>-7.8[80]	
[81]	*In vitro*	*Alpinia zerumbet* (Pers.) B.L.Burtt & R.M.Sm.	Proanthocyanidins[81]	Isolation: Gel permeation chromatography<br><br>Characterisation: HPLC<br><br>Assay: Cytopathic effect assay	Influenza A virus<br><br>Porcine epidemic diarrhoea virus	Proanthocyanidins extracted from *A. zerumbet* (AzPACs) showed antiviral activity against influenza A virus by decreasing the viral titre by >3 log at 0.1 mg/mL. AzPACs also remarkably inhibited porcine epidemic diarrhoea virus (PEDV) by decreasing its titre by >4 log at 0.1 mg/mL.	AzPACs effectively inactivated the influenza A virus and PEDV *in vitro* in a dose-dependent manner. Therefore, AzPACs possess broad-spectrum antiviral activity.
[72]	*In silico*	*Amomum nilgiricum* V.P.Thomas & M.Sabu	Serverogenin acetate[58]	Characterisation: GC-MS<br><br>Assay: Molecular docking	NA	Serverogenin acetate formed hydrogen bonds with Gln91 in the HIV-1 reverse transcriptase (1rev) viral target protein. The docking results revealed a binding energy of -3.65 kcal/mol, a ligand efficiency of -0.09 kcal/mol, an inhibition constant of 2.11 µM and van der Waals with hydrogen bonding and desolvation energy of -4.32 kcal/mol.	The binding affinity of serverogenin acetate to the binding sites of 1rev suggests its antiviral activity.

Note: NA = not available; TLC = thin-layer chromatography; GC-MS = gas chromatography-mass spectrometry; LC-MS/MS = liquid chromatography with tandem mass spectrometry; NMR = nuclear magnetic resonance; LC-MS = liquid chromatography-mass spectrometry; HPLC = high-performance liquid chromatography; GC-MS = gas chromatography-mass spectrometry

The superscript numbers reported in the Supplementary Tables correspond to the bold numbers in the main text for each compound.

DISCLAIMER: The above article has been published, as is, ahead-of-print, to provide early visibility but is not the final version. Major publication processes like copyediting, proofing, typesetting and further review are still to be done and may lead to changes in the final published version, if it is eventually published. All legal disclaimers that apply to the final published article also apply to this ahead-of-print version.
